# Cellular Base of Mint Allelopathy: Menthone Affects Plant Microtubules

**DOI:** 10.3389/fpls.2020.546345

**Published:** 2020-09-16

**Authors:** Mohammed Mahmood Sarheed, Fatemeh Rajabi, Maritta Kunert, Wilhelm Boland, Sascha Wetters, Kai Miadowitz, Andrzej Kaźmierczak, Vaidurya Pratap Sahi, Peter Nick

**Affiliations:** ^1^Molecular Cell Biology, Botanical Institute, Karlsruhe Institute of Technology, Karlsruhe, Germany; ^2^Department of Bioorganic Chemistry, Max Planck Institute for Chemical Ecology, Jena, Germany; ^3^Department of Cytophysiology, Faculty of Biology and Environmental Protection, University of Łódź, Łódź, Poland

**Keywords:** microtubules, monoterpenes, allelopathy, menthone, mint

## Abstract

Plants can use volatiles for remote suppression of competitors. Mints produce essential oils, which are known to affect the growth of other plants. We used a comparative approach to identify allelopathic compounds from different Mints (genus *Mentha*, but also including Cat Mint, *Nepeta cataria*, and Corean Mint, *Agastache rugosa*, belonging to sisters clades within the *Mentheae*) using the standard cress germination assay as readout. To understand the mechanism behind this allelopathic effect, we investigated the response of tobacco BY-2 cell lines, expressing GFP-tagged markers for microtubules and actin filaments to these essential oils. Based on the comparison between bioactivity and chemical components, we identified menthone as prime candidate for the allelopathic effect, and confirmed this bioactivity targeted to microtubules experimentally in both, plant cells (tobaccoBY-2), and seedlings (*Arabidopsis thaliana*). We could show that menthone disrupted microtubules and induced mortality linked with a rapid permeabilization (less than 15 min) of the plasma membrane. This mortality was elevated in a tubulin marker line, where microtubules are mildly stabilized. Our study paves the way for the development of novel bioherbicides that would be environmentally friendly.

## Introduction

Plants have developed mechanisms to defend and adapt themselves against biotic and abiotic stress factors. As part of this defense, plants can suppress the growth of competing neighbors by releasing chemicals, a process called allelopathy (Lambers et al., 2008). Allelochemicals can be produced in different organs and can suppress the growth of target plants by different mechanisms upon contact of the allelochemicals with the target plants (Choesin and Boerner, 1991). Plant essential oils, often with monoterpenes as primary components, accumulate in different vegetative organs, such as leaves, bark, wood, roots, but also in flowers or fruits, sometimes in specialized glands, sometimes in lysogenic or schizogenic oil ducts (Bruneton, 1995). In order to exert their effect, they have to be released into the environment, which is the reason, why many allelochemicals are volatiles. This leads to an interesting question that has been rarely addressed: how does the source plant prevent that it is not inhibited by its own allelochemicals? One possibility to obtain specificity would be to use compounds that activate a signaling process in the receiving plant that is not activated in the donor plant itself, due to inactivated binding of the compound.

Since allelopathy is specific, it offers interesting applications for biological pest control. In fact, plants producing essential oils have been used extensively for growth regulation, herbicidal, insecticidal, or other agricultural applications (Bajalan et al., 2013; Cheng and Cheng, 2015; Skrzypek et al., 2015). Biocontrol by essential oils has a long history—for instance, these oils were the first food preservatives used by humans (Thompson, 1989), and often already the crude extracts have been found to exert efficient antifungal, antimicrobial, cytostatic, and insecticidal activities (Sivropoulou et al., 1995; Mkaddem et al 2008). In some cases, the bioactivity has already been broken down to individual compounds. For instance, oil from *Artemisia scoparia* can inhibit germination and seedling growth of three weeds, and this allelopathic effect could be attributed to its major constituent, β-myrcene (Singh et al., 2009). Components from *Eucalyptus* essential oil, such as eucalyptol, α- and β-pinene, camphene and camphor were reported to inhibit cell proliferation in roots by interfering with DNA synthesis in organelles and nucleus of the *Brassica campestris* root meristem (Nishida et al., 2005). Monoterpenes (camphor and menthol) have been found to affect stomatal opening through cytoskeletal changes as a response to the monoterpenes and have effects on the growth of *Arabidopsis thaliana* seedlings (Kriegs et al., 2010). Similarly, citral was found to be a strong inhibitor of seed germination in *Triticum sativum*, *Amaranthus palmeri* and *Brassica nigra* (Dudai et al., 1999). However, in many cases, it has remained unclear which compound or which mixture of compounds is responsible for the alellopathic effect of a given essential oil, such that the mechanisms behind the specificity of allelopathy have remained fairly elusive.

One strategy to address the specificity of bioactive compounds is to compare related species that differ in their allelopathic effect. So called aromatic plants represent an interesting target, and are known to emit volatile growth inhibitors (Muller and Muller, 1964; Weir et al., 2004). Among the aromatic plants, the Lamiaceae are of particular interest, because this family which typically produces a broad repertory of terpenoids and phenolic compounds (Wink, 2003) is extremely diverse with estimated 7,000 species that often differ in their chemical characteristics, and are grouped into several hundreds of genera (Harley et al., 2004). The pronounced chemical diversity in this group indicates that chemical signaling directed to other organisms might have been an important driving force in speciation, such that there is a good chance to identify specific allelochemicals.

Within the Lamiaceae, the Mints (*Mentha*) are of economical relevance, and have a long history of human use as aromatic and medical compounds. This genus comprises around 30 species of aromatic perennial herbs, distributed mostly in temperate and sub-temperate regions of Eurasia, Australia, Africa, and North America (Dorman et al., 2003; Kanatt et al., 2007; Hui et al., 2010). Recently, the chemical diversity of this genus has been made accessible by genomics defining a repertory of metabolic genes that have contributed to the different chemotypes (Mint Evolutionary Genomics Consortium, 2018).

Commercial use is concentrated around Peppermint (*M. x piperita*), Spearmint (*M. spicata*), and Corn Mint (*M. canadensis*). While for the first two species, often leaf material is used, for instance as herbal tea, corn mint is exclusively cultivated for oil production (Small, 1997; Oudhia, 2003). The main compounds of *Mentha* essential oils are monoterpenes like limonene, menthone, and menthol, which are synthesized in glandular trichomes on the leaf surface (Turner et al., 2000). Although coming from closely related species, the essential oils from the different Mints show characteristic differences in composition: While the economically dominating essential oil from Peppermint (*M. x piperita*) contains menthol and menthone as main components (Gul, 1994), Corn Mint (*M. canadensis*) is interesting as the richest source of natural menthol (Sharma and Tyagi, 1991; Shasany et al., 2000), while Spearmint (*M. spicata*) is rich in carvone and therefore widely used as spice in several countries (Kokkini et al., 1995).

The differences of oil composition in these closely related species are linked with different bioactivities that are often of medical interest: The oil of Spearmint (*M. spicata*) is a natural antioxidant (Kanatt et al., 2007), while an extract from Korean Mint (*Agastache rugosa*), another member of the Lamiaceae family, inhibits HIV integrase, a protein responsible for integrating the viral DNA into the human genome, and therefore being relevant as a possible target for selective antiviral therapy (Kim et al., 1999). Likewise, essential oils extracted from the mint species *M. pulegium* and *M. spicata*, containing mainly pulegone, menthone, and carvone, showed strong insecticide activity on *Drosophila melanogaster*, whereby the oil of *M. spicata* was, in addition, mutagenic. Here, pulegone was found to responsible for the activity as insecticide, while the genotoxic activity could be attributed to menthone. Interestingly, the strong toxicity of pulegone was suppressed in the presence of menthone, demonstrating that synergistic or antagonistic interactions between compounds of a given oil are relevant for bioactivity (Franzios et al., 1997).

The inhibition of root growth (as well as the genotoxicity) indicates that menthone targets to microtubules. In fact, microtubules are known to be affected by important herbicide classes, such as the dinitroaniline herbicides (Anthony et al., 1998). Disassembly of mitotic microtubules, by either anti-microtubule drugs or mutations, disrupts mitotic activity, culminating in asymmetries of newly formed cell plates (Yoneda et al., 2005; Kawamura et al., 2006; Vaughn, 2006). Likewise, disassembly of cortical microtubules interferes with the ordered deposition of cellulose microfibrils, such that cell elongation is blocked. Actually, it was the loss of expansion axiality after treatment with colchicine that brought Paul Green to predict the existence of “microtubules” (Green, 1962), and, one year later, led to the discovery of “microtubules” by Ledbetter and Porter (1963). Disruption of plant microtubules by monoterpenes has already been shown for citral, which can inhibit the expansion growth of *Arabidopsis* roots by affecting cortical microtubules (Chaimovitsh et al., 2010), and also impair the proliferation of tobacco BY-2 cells, accompanied by a high frequency of asymmetric cell walls (Chaimovitsh et al., 2012). Taxol, a compound which is stabilizing microtubules, was able to protect BY-2 cells from this citral-mediated microtubule disruption, indicative of microtubules as primary targets of citral. Allelopathic effects of Mint have been described for Horse Mint (*M. longifolia*, wheat seedlings, Bajalan et al., 2013) and Peppermint (*M. x piperita*, sunflower seedlings, Skrzypek et al., 2015).

The current study ventured to identify compounds underlying Mint allelopathy and to address their cellular mode of action. This was designed as comparative approach, where the differential bioactivity of different Mint oils was used as logical filter to identify active compounds. This work is built upon three axes: (1) to ensure the validity of our findings, the biodiversity used as input for the approach had to be authenticated, (2) based on this validated biodiversity, a candidate compound correlated with bioactivity could be identified as menthone, (3) menthone-induced elimination of microtubules could be identified as cellular mode of action underlying the allelopathic effect.

## Material and Methods

### Plant Material

The plants, which were used for oil extraction, were raised at the Botanical Garden of the Karlsruhe Institute of Technology, Karlsruhe, Germany, in glass houses. To boost the formation of essential oils, the plants were exposed to sunlight for a few days prior to extraction. Plants of different species were grown in separate pots. The identity of the accessions was verified by taxonomic identification, as well as by genetic barcoding (**Table 1**).

**Table 1:** Accessions used in the current study maintained as living specimens in the Botanical Garden of the Karlsruhe Institute of Technology under the indicated voucher ID.

KIT ID	Declared taxonomy	Vernacular names	Source	GenBank ID for *psbA-trnH igs*	Identity
7579	*Mentha spicata* L.	Spear Mint	BG KIT	MH753576	*M. spec*.
5391	*Mentha spicata* L. var. *crispa*	Curly Mint	BG KIT	MH753570	*M. spicata* L.
8680	*Mentha aquatica* L.	Water Mint	WEL 3/451	MH753578	*M. aquatica* L.
8681	*Mentha arvensis* L.	Corn Mint	WEL 3/437	MH753577	*M. spec*.
8682	*Mentha longifolia* (L.) L.	Horse Mint	WEL 3/72	MH753572	*M longifolia* (L.) L.
5393	*Mentha x piperita* L.	Pepper Mint	BG KIT	MH753571	*M. x piperita* (L.)
3638	*Mentha suaveolens* Ehrh.	Apple Mint	BG Vakratot	MH753574	*M. spec*.
4643	*Nepeta cataria* L.	Catnip	BG KIT	MH753573	*N. cataria* L.
4639	*Melissa officinalis* L:	Common Balm	BG KIT	MH781964	*M. officinalis* L:
7576	*Agastache rugosa* (Fisch. & C.A.Mey.) Kuntze	Korean Mint	BG KIT	MH753575	*A. rugosa* (Fisch. & C.A.Mey.) Kuntze

**Figure 1:**
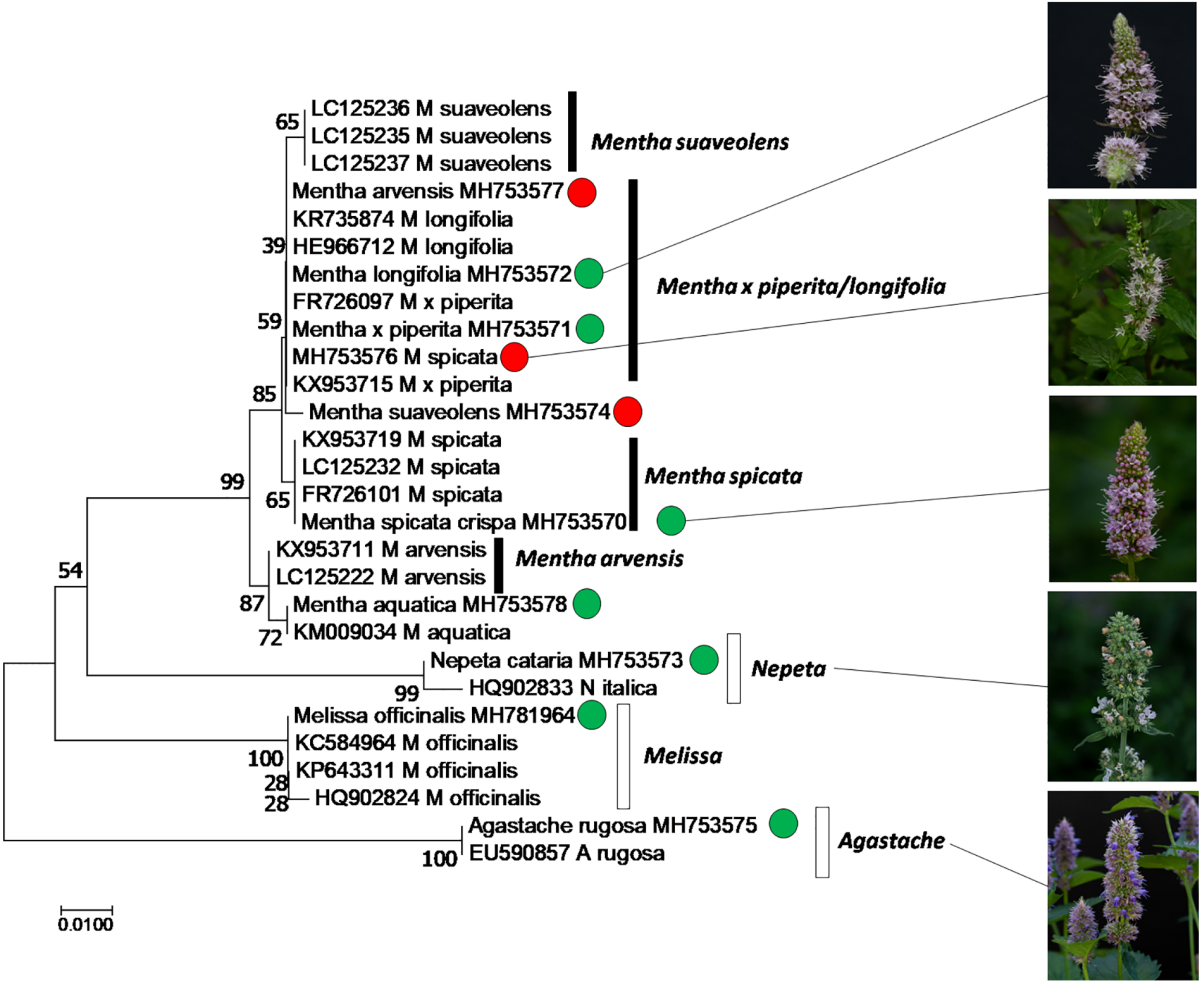
Phylogenetic relationship of the accessions used in this study, inferred from the marker *psbA-trnH intergenic spacer* by the Neighbor-Joining algorithm using 28 sequences from different species of Mentha (black boxes), along with neighboring taxa and the outgroup *Melissa* (white boxes). Green circles represent accessions used in this study, where the declared identity was confirmed, based on sequences, red circles indicate accessions that turned out to be mis-assigned.

Scientific taxonomy follows the The Plant List (www.theplantlist.org). All psbA-trnH igs sequences from these accessions were deposited in GenBank under the given ID and identity verified by comparing these accessions with curated sequences from GenBank (see **Figure 1**).

### Genetic Identification

Fresh leaves of reference plants (~60 mg) were used for DNA extraction, the DNA was isolated using the Invisorb^®^ Spin Plant Mini Kit (Stratec biomedical AG) following the protocol of the producer. The weighed plant material was shock-frozen in liquid nitrogen and homogenized in reaction tubes with steel beads (QiagenTissueLyser) for 2 min. The isolated DNA was evaluated by spectrophotometry (Nanodrop, Peqlab) for quality and quantity, and DNA concentration was diluted to 50 ng/µl as template for genomic PCR. A reaction volume of 30 µl containing 20.4 µl nuclease free water (Lonza, Biozym), 1-fold Thermopol Buffer (New England Biolabs), 1 mg/ml bovine serum albumin, 200 µM dNTPs (New England Biolabs), 0.2 µM of forward and reverse primers (**Table 2**), 100–150 ng DNA template, and 3 units of Taq polymerase (New England Biolabs) was used to amplify the marker sequences. The *psbA-trnH intergenic spacer* region was amplified in a thermal cycler by initial denaturation at 95°C for 2 min; followed by 33 cycles at 94°C for 1 min, annealing at 56°C for 30 s, and elongation at 68°C for 45 s; ending with an extension of 68°C for 5 min. The PCR amplicons were evaluated by agarose gel electrophoresis using NEEO ultra-quality agarose (Carl Roth, Karlsruhe, Germany). The DNA was visualized using SYBRsafe (Invitrogen, Thermo Fisher Scientific Germany) under blue light excitation (Invitrogen, Safe Imager λ = 470 nm). A 100-bp size standard (New England Biolabs) was used to determine the fragment size of the products. The amplicons were purified for sequencing using the MSB^®^ Spin PCRapace kit (Stratec). Sequencing was conducted by Macrogen Europe (Netherlands) or GATC (Germany), and the quality of the obtained sequences was tested by FinchTV Version 1.4.0 (https://digitalworldbiology.com/FinchTV). The marker region was sequenced from two directions to get a robust result. The resulting two sequences were merged for each accession by aligning the forward read with the reverse complemented backward read. For sequence alignment and phylogenetic analysis, the program MEGA7 (Version 7.0.14) with the integrated tree explorer was used. The sequences were aligned using the Muscle algorithm of MEGA7. Alignments were trimmed to the first nucleotide downstream of the forward primer and the nucleotide preceding the reverse primer. With the same software, the phylogenetic relationships were inferred by using the neighbor-joining algorithm with a bootstrap value that was based on 1,000 replicates. The species *Melissa officinalis* was chosen as an outgroup. Additional *psbA-trnH intergenic spacer* sequences for the use in molecular phylogeny were obtained from GenBank using a BLAST tool in combination with the Taxonomy View tool.

**Table 2:** Oligonucleotide primers used for genetic identification of the tested accessions.

Name	5’ → 3’ sequence	Target	Reference
*psbA^u^*	GTTATGCATGAACGTAATGCTC	psbA-trnH intergenic spacer	Sang et al., 1997
*trnH^u^*	CGCGCATGGTGGATTCACAATCC		Tate and Simpson, 2003

### Oil Extraction

To extract the essential oils for experiments with living plant cells and entire plants, the essential oil was extracted by a simple water-steam distillation method. For each plant species, 30–50 g of fresh leaf material were harvested, frozen in liquid nitrogen, and mildly ground to break up the oil cavities of the glandular hairs. The frozen plant particles and a stirrer bar were added to the still pot, which was then filled until the level of water reached the edge of the heating mantle. The distillation apparatus was filled with distilled water and the water cooling system was connected in a way that enables water flow in the opposite direction of the still. The oil was now distilled by heating the plant material for about two hours to 100°C. The volume of the extracted oil was collected in a graded burette, and the oil phase measured, and, to optimize yield, the tube was washed with a small quantity of n-hexane. Afterward the distillate was sealed in a glass vial and stored at 4°C till experimental use. The oil was later used for various treatments.

### GC-MS Analysis

The essential oils were analysed by GC-MS analysis at the Max Planck Institute for Chemical Ecology Jena. For GC-MS, a TRACE MS (Thermo-Finnigan) device equipped with a ZB5 column (15 m, 0.25 mm I.D, 0.25 µm film thickness) was used with a 10-m guard column (Phenomenex, Aschaffenburg, Germany). Mass spectra were collected using the electron impact (EI) mode at 70 eV, 33–450 m/z. Volatiles were eluted under programmed conditions: 40°C (2 min isotherm), followed by heating at 10°C min^-1^ to 220°C and, further, at 30°C min^-1^ to 280°C, using helium (1.5 ml. min^-1^) as carrier gas. The GC injector (split ratio 1:7), transfer line and ion source were set at 220, 280, and 200°C, respectively.

### Cell Culture

Different strains of tobacco BY-2 (*Nicotiana tabacum* L. cv Bright Yellow-2) cell lines (Nagata et al., 1992) were used for this study. Cells were sub-cultured in Murashige-Skoog medium at weekly intervals, by inoculating 1.5 ml of stationary culture cells into 30 ml of fresh new medium. The cells were cultivated at 26°C in the dark under constant shaking as described previously (Maisch and Nick, 2007). To follow microtubule responses *in vivo*, the strain BY2-TuB6-GFP, expressing β-tubulin (AtTUB6) fused to GFP under the control of the constitutive Cauliflower Mosaic Virus 35S promotor was used (Hohenberger et al., 2011), while the responses of actin filaments were followed using the strain GF11, expressing the second actin-binding domain of fimbrin (AtFIM1) in fusion with GFP, also under a CaMV-35S promoter (Sano et al., 2005).

### Live-Cell Imaging

The response of the cells to the extracted oils, but also to pure candidate compounds identified in those oils was followed by live-cell imaging. The information on purity and sources for these compounds is given in **Table 3**. After three days of cultivation, about 1 ml of cell suspension culture were transferred to a tube and diluted with cultivation medium to a cell density appropriate for microscopic observation. Slides for microscopy were mounted prepared as follows (**Supplementary Figure S4A**): a small volume (20 μl) of cell suspension was spread on the slide. Subsequently, close to the cells, but without touching them, 0.5 μl of oil or pure compounds were added on both sides, before a 40 x 24 mm coverslip was placed on the slide. Thus, there was no direct contact between oil and suspension, such that the action of the compounds in the essential oils had to occur through the gas phase. Although the essential oils were not diluted, they contained traces of *n*-hexane from the purification process. Therefore, solvent controls with the corresponding volume of *n*-hexane were included. Among the candidate compounds, menthol was solid and was dissolved in 10% in ethanol, therefore, ethanol was included as further solvent control. The cells were observed under the Zeiss Cell Observer Spinning Disc (Zeiss, Jena, Germany) equipped with a cooled digital CCD camera (AxioCamMRm), and a spinning-disc device (YOKOGAWA CSU-X1 5000) microscope, using a 20x air, and a 63x oil immersion objective, respectively. The cells were observed under DIC (differential interference contrast (DIC), and after excitation with the blue line (488 nm) of an Ar-Kr laser (Zeiss, Jena, Germany) to detect the GFP signal. For each oil or compound, and each cell line, cells were chosen randomly and followed for 30 min.

**Table 3:** Purity and source information for the monoterpene compounds used in the current study.

compound	purity (%)	source	catalogue number
Geraniol	analytical standard	Sigma-Aldrich	163333
β-Citronellol	95	Sigma-Aldrich	C83201
α-Pinene	98	Sigma-Aldrich	147524
β-Pinene	pure	Roth	7106.1
Linalool	97	Sigma-Aldrich	L2602
Menthone	analytical standard	Merk	95401
DL-Menthol	analytical standard	Sigma-Aldrich	05174
R-(+)-Limonene	analytical standard	Sigma-Aldrich	62118

### Cell Mortality Assays

To test the effect of the oils on the overall viability of the cells, the Evans Blue Dye Exclusion Assay (Gaff and Okong’ O-Ogola 1971) was used. Viable cells exclude the dye from their cytoplasm and remain unstained, while dead or dying cells, due to loss of membrane integrity, take up the dye (Tisserant et al., 2014). The effect of the essential oils was assessed in cells with high mitotic activity cells (day 3 after subcultivation) in sampling aliquots. For this purpose, 500 μl of cell suspension were transferred to a reaction tube, and incubated with 0–1 μl of the respective essential oil or compound, for either 15 or 30 min. After incubation, the suspension was transferred to a custom-made cell filter tube (Nick et al., 2000). Based on preparative experiments with different staining and rinsing times, a protocol was defined with staining for 5 min in 2.5% Evans Blue, before rinsing the cells in BY-2 cultivation medium three times for 2 min each. Subsequently, 20 μl of the suspension were transferred into onto a hemato-cytometer slide (Fuchs-Rosenthal) and scored under the bright-field microscope. The percentage of observed cells that were unstained was calculated and used as readout for viability. Data represent three independent replications with a population of 600 individual cells per replication.

To obtain a more detailed insight into the mortality induced by essential oil of *A. rugosa*, menthone/isomenthone, or limonene, a double labelling with the membrane permeable dye Acridine Orange and the impermeable dye Ethidium Bromide was conducted (Doniak et al., 2016). Aliquots of freshly harvested tobacco BY-2 cells (200 µl) were mixed with the respective compound and incubated for 15 min in a closed reaction tube in the dark at 25°C on an orbital shaker (IKA^®^KS 260 basic). Then, a sample of 50 µl was stained with the same volume of staining mixture (100 μg.ml^-1^Acridine Orange, and 100 μg.ml^-1^ Ethidium Bromide in a 0.1 M sodium phosphate buffer at pH 7.4) for 5 min. After staining, the sample was diluted with 100 µl of the same buffer, and 50 µl of the mixture transferred to a glass slide, covered with a coverslip and observed by fluorescence microscopy (Diaplan, Leitz) with excitation in the blue (filter set I3, excitation 450–490 nm, beam splitter 510 nm, emission filter >515 nm) using a digital acquisition system (Leica DFC 500) and software (Leica Application Suite, v4). The nuclei of cells with tight membranes will appear green, because they are exclusively labelled by Acridine Orange. With progressive permeabilization of the membrane, the red signal from Ethidium Bromide will increase, such that the nuclei turn over orange into red. This color change was quantified and classified as described in Byczkowska et al. (2013). Each frequency distribution represents around 250–300 nuclei in each of two experimental series.

### Cress Germination Assay

In order to quantify allelopathic activity of essential oils, or candidate compounds identified therein, the standard cress germination test (International Seed Testing Association, www.seedtest.org) was performed in Plexiglass boxes (30 mm x 20 mm x 15 mm) that were covered and sealed. The bottom of the boxes was covered with humidified absorbent papers (No. 914446/4; Hartmann, Heidenheim/Brenz, Germany). Hundred seeds of *Lepidium sativum* (var. ‘Glatt’, FloraSelf Co.) per box (30 mm x 20 mm x 15 mm) were sown equidistantly, and a glass slide with filter paper loaded with a defined volume (0.1, 1, or 10 µl corresponding to 0.01, 0.1, or 1 ppm) of the respective compound or the respective solvent was placed in the center of the box. After mounting, the box was sealed with Parafilm, and stored in an incubator with a temperature of 27°C in the dark for two days. After two days, the germinated seedlings were counted, and hypocotyl and root length measured. Each experiment was replicated three times. The resulting dose-response curves were fitted using a sigmoidal model.

### Visualization of Microtubules in *Arabidopsis thaliana*

The effect of oil extracted from *A. rugosa* along with menthone was assessed on both, cotyledons and hypocotyls of etiolated *Arabidopsis thaliana* seedlings, where either microtubules were labelled with GFP. The line GFP-TuB6 over-expressed a N-terminal fusion of β-tubulin 6 from *A. thaliana* N-terminally fused with GFP under a Cauliflower Mosaic Virus (CaMV) 35S promoter generated in the Col-0 background (Nakamura et al., 2004) was kindly provided by Dr. Kateřina Schwarzerová, Institute of Plant Physiology, Charles University, Prague. The seeds were grown on moist filter paper in a petri dish at 25°C for 5 days in the dark. To assess the effect on the cytoskeleton, 50 µl of either essential oil extracted from *A. rugosa*, or pure menthone were transferred onto a microscopic glass slide. The seedlings were placed directly in the respective liquid, covered by a coverslip, and then the sample was immediately observed by spinning disc microscopy. As negative control, plants were placed in distilled water instead. In a supplementary experiment, the compounds were administered through the gas phase analogous to the time-lapse studies on tobacco BY-2 cells (**Supplementary Figure S4B**).

## Results

### Verification of Taxonomic Identity

In order to authenticate the plants used in the study, we used the *psbA-trnH intergenic spacer*, a plastidic marker. The sequences obtained from the amplified regions were aligned with their curated homologues recovered from GenBank using a BLAST search and the Taxonomy View tool. Only curated sequences from sources, where the identity of the plant had been determined, were used for reference (i.e. sequences from commercial products were excluded, since they are often of doubtful identity). Based on the aligned sequences, a phylogenetic tree was constructed and bootstrapped using the neighbor-joining algorithm (**Figure 1**). The tree clearly shows that the sequences from *Mentha* species clustered separately from sequences obtained from the other tested genera within the Lamiaceae family (*Agastache*, *Nepeta*, *Melissa* that all formed well supported separate clades). This *Mentha* clade differentiated into four subclades: *M. arvensis* and *M. spicata* were clearly distinct with sufficient bootstrap support (99% for both clades against the other *Mentha* accessions, 87% for the separation between *M. arvensis* and *M. spicata*). Also *M. suaveolens* differentiated into a separate clade, albeit with lower bootstrap support (65%). The largest group comprised *M. longifolia* and *M. x piperita*, without any differentiation between these two species. Our analysis also revealed that some plants had been wrongly assigned. We can clearly see that MH753577, declared as *Mentha arvensis*, MH753576, as *Mentha spicata*, and MH753574, declared as *Mentha suaveolens* cluster with *Mentha longifolia* and *Mentha x piperita*. These plants, which had obviously been mis-identified, were then annotated as *Mentha spec*. (**Table 1**).

### Identification of Menthone as Germination Inhibitor

**Figure 2:**
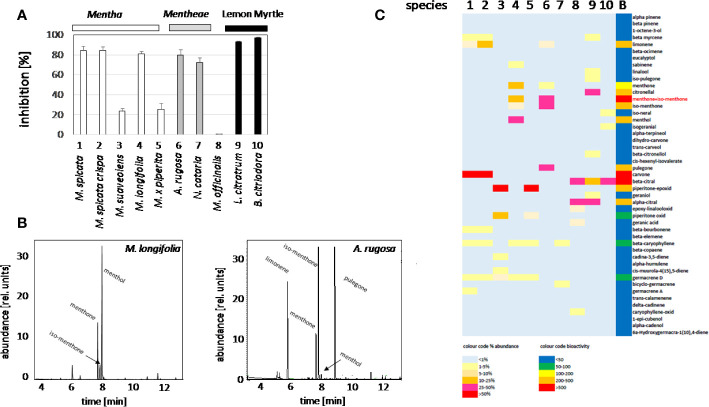
Activity Guided Fractionation of oils extracted from different mint plants. **(A)** Inhibition of germination by essential oils extracted from different Lamiaceae. Inhibition (%) of cress seed germination after 48 h incubation in darkness at 27°C in presence of essential oils from differentMentha and other Lamiaceae accessions. The effect of essential oil from the two species of Lemon Myrtle is shown as a positive control. Data represent means and SE from three independent experimental series (n = 100 seedlings). **(B)** Representative gas chromatography profiles for the essential oils from *M. longifolia* and *A. rugosa*. **(C)** Heat map for relative abundance of compounds and bioactivity scores (as defined in the method part).

In order to identify candidate compounds responsible for the bioactivity of the plant oils, the cress standard bioassay for seed germination was used, followed by an analysis of the GC profiles of the extracted plant oils. Based on preparatory studies, 0.1 ppm of essential oil extracted from different accessions, were employed, since they produced a substantial inhibition for some oils (**Figure 2A**). The strongest inhibition of seed germination was observed for the oils from *M. spicata* (in fact just confirmed as *M. spec*.)*, M. spicata crispa, M. longifolia, A. rugosa*, and *N cataria*, while the oil from *M. suaveolens* (just confirmed as *M. spec*.), *M. x piperita*, and *Melissa officinalis* inhibited cress germination only mildly. As positive control, we used two essential oils obtained from two species designated as Lemon Myrtle (*Leptospermum citratum* and *Backhousia citriodora*), because these are rich in citral, a compound with known allelopathic activity (Chaimovitsh et al., 2010). As expected, also these positive controls exerted a stringent inhibition. In order to get insight into the chemical compounds responsible for this bioactivity, the oils from these ten accessions were analyzed by GC, and many peaks identified and measured using commercially available standards (**Supplementary Data S1**). These profiles were then compared with the observed bioactivity. To quantify the average bioactivity for a given compound, a bioactivity score was defined as

$$B=\frac{1}{n}[\Sigma a(i)*g(i)]$$

**Figure 3:**
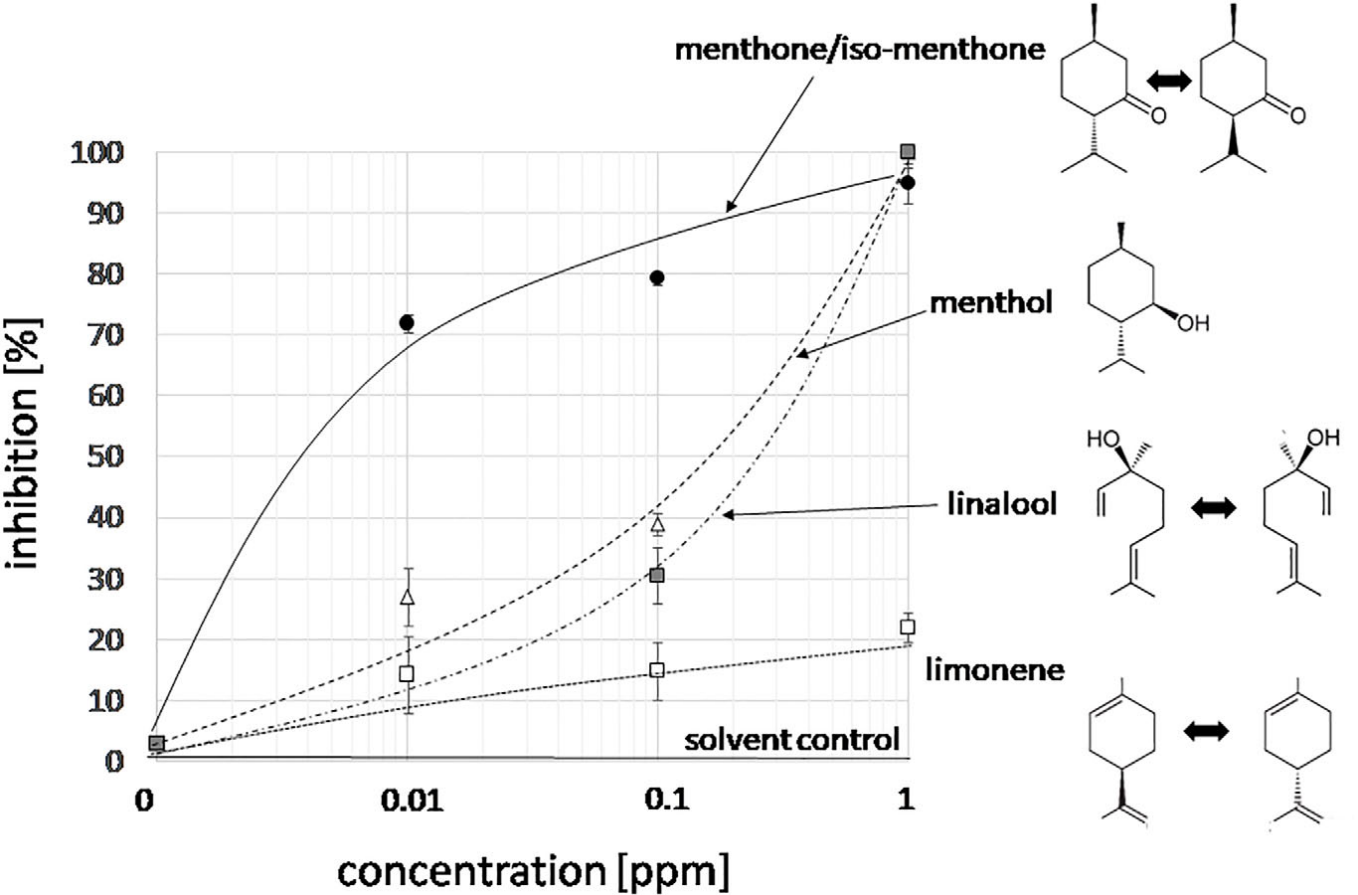
Dose-response relation for the germination inhibition of different monoterpene compounds specific to essential oils from Lamiaceae in the cress assay scored after 48 h of incubation in darkness at 27°C in presence three concentrations of menthone/isomenthone, menthol, linalool and limonene. The effect of the two solvent controls (0.1 ppm n-hexane, or 0.1 ppm ethanol) is shown by the solid line. Data represent means and SE from three independent experimental series (n = 100 seedlings).

With *n* number of accessions (10), *a(i)* relative peak area of the respective compound for accession *i*, and *g(i)* the germination inhibition observed by the essential oil of accession *i*. This bioactivity score increased with the relative abundance of the respective compound in the oil, and with the bioactivity of the essential oil. If an essential would be composed only of one compound and this compound would give full inhibition, a bioactivity score of 10,000 would result. The largest scores obtained in this assay were around 1,000 (**Supplementary Data S1**). From this analysis, three candidates emerged (**Figure 2C**): menthone/isomenthone (since both compounds interconvert easily by enolization, they were pooled), β-citral (isomeric with α-citral), and carvone. Citral has already been described as allelopathic compound with anti-microtubular activity (Chaimovitsh et al., 2010), and, thus, served as a positive control for the feasibility of the approach, carvone, while being the most active compound, was mainly found in an accession of unclear identity (*M. spicata*, which in fact clustered with *M. x piperita*). We focused therefore on the third compound, menthone/isomenthone, which was most abundant in *M. longifolia* and in *A. rugosa*. The comparison of GC profiles for the oils from *M. longifolia* and *A. rugosa* (**Figure 2B**) show in both cases abundance of menthone and isomenthone, but also clear differences in the chemical speciation: while the overall content of menthone and isomenthone was comparable in both species (around 30% relative peak area), *M. longifolia* harbours menthol as main component (more than 50% relative peak area), which is barely detectable in the essential oil from *A. rugosa*. Instead, *A. rugosa* oil contains significant amounts of the menthone precursors limonene and pulegone. Based on the abundance of compounds and the high bioactivity score of menthone, we determined dose-response relationships for the inhibition caused by menthone/isomenthone, menthol as their derivative, limonene as their precursor, and linalool as chemically similar monoterpene with differing structural features (**Figure 3**). In order to account for possible effects of solvents, 0.1 ppm n-hexane, and 0.1 ppm ethanol were used as solvent controls. Both had only negligible effect on the germination of seeds. A dose-dependent effect on germination was observed when 0.01, 0.1, and 1 ppm of menthone/isomenthone, menthol, linalool, and limonene were used (**Figure 3**). The strongest effect can be seen with menthone/isomenthone, where even a low concentration of 0.01 ppm inhibits the germination by about 70%, which rose to almost 99% at a concentration of 1 ppm. The least effects were observed for limonene, where even with a high concentration of 1 ppm the inhibition was just 20%. Menthol and linalool produced a similar pattern, where a dose-dependent gradual increase could be seen. The shape of the fitted sigmoidal curves differed. For concentrations up to 0.1 ppm, both, menthol and linalool inhibited only mildly (between 30 and 40%, as compared to 80% for menthone/isomenthone). Only at the highest concentration, 1 ppm, they reached to the level seen for menthone/isomenthone. Thus, menthone/isomenthone emerged as most promising candidate for the inhibitory effect of *M. longifolia*, and *A. rugosa* essential oils, while their structurally similar precursor limonene, as well as their structurally similar derivative menthol, was much less active.

### Menthone/Isomenthone Are Cytotoxic, depending on Tubulin

**Figure 4:**
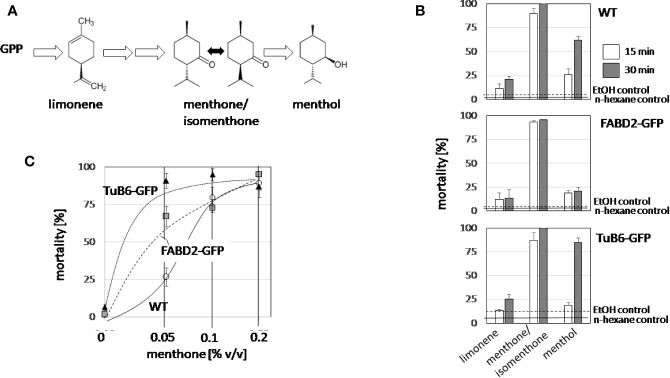
Structure-function relationship for the cytotoxicity of limonene, menthone, and menthol. **(A)** Structures and position of the tested compounds in the biosynthetic pathway for menthol. **(B)** Time course of cytotoxicity in non-transformed BY-2 cells (WT), in cells overexpressing GFP fusions with the actin-binding domain 2 of fimbrin (actin), and in cells overexpressing AtTuB6 (microtubules). The values observed for the solvent controls with EtOH (0.2% v/v) after 30 min are represented by the dashed lines, the values observed for the solvent controls with n-hexane (0.2% v/v) after 30 min by solid lines. **(C)** The dose response of cytotoxicity over the volume of menthone in the assay for the three cell lines, scored after 15 min. Data represent mean and standard errors from three independent experiments scoring 600 individual cells per data point.

To get insight into the mode of action of the bioactive candidate compounds identified in the essential oils, we probed for the potential cytotoxicity of menthone/isomenthone (the commercially available product was a racemate of around 80% menthone and 20% isomenthone), its precursor limonene, and its derivative menthol (**Figure 4A**), along with 0.2% v/vn-hexane, and 0.2% v/v ethanol as solvent controls in tobacco BY-2 cells. Mortality was scored using the Evans Blue Dye Exclusion test in a time-course experiment, testing mortality at 15 or 30 min after addition of 1 µl (corresponding to 0.2% v/v) of the respective compound (**Figure 4B**). To test a potential impact of the cytoskeleton, in addition with the non-transformed BY-2 cells (WT), two cytoskeletal marker lines were tested: the actin marker line GF11, where actin is labelled by overexpression of the actin binding domain 2 of fimbrin with GFP (FABD2-GFP), and the microtubule marker line TuB6, where microtubules are labelled by overexpression of *Arabidopsis thaliana* β-tubulin 6 fused to GFP (TuB6-GFP). In non-transformed BY-2 cells (**Figure 4B**, top), menthone/isomenthone induced a rapid increase of mortality, already after 15 min, more than 90% of the cells were dead. In contrast, limonene was mostly ineffective with only 20% mortality reached after 30 min, menthol exerted intermediate cytotoxity, which developed slower (at 15 min, only 25% of the cells were killed, while at 30 min, mortality had increased over 60%), and the solvent control did not produce any significant mortality. The time course was almost identical in the actin-marker line (**Figure 4B**, centre). For the microtubule-marker line, the response to limonene was basically unaltered, and the response to menthone/isomenthone, which was already saturated in the non-transformed WT, was still close to saturation. A clear difference was observed for the response to menthol, however (**Figure 4B**, bottom): here, the amplitude of the response was significantly amplified by around the half to reach almost 90% mortality after 30 min. Since the concentration of menthone/isomenthone in the time-course experiment was close to saturation, we conducted then a dose-response study with lower concentrations (**Figure 4C**). This experiment revealed clearly that the sensitivity of the microtubule marker line was elevated, because saturation was reached at around 4-fold lower concentrations of menthone/isomenthone as compared to the wild type. Interestingly, also the actin marker line was somewhat more sensitive than the non-transformed WT, albeit not to the same extent.

To get more insight into the type of cell death induced by menthone/isomenthone, its precursor limonene, and the essential oil of *A. rugosa*, a double-staining strategy using two fluorescent dyes differing in their membrane permeability was administered (Doniak et al., 2016). Since one of these dyes, Acridine Orange, permeates the plasma membrane, while the other dye, Ethidium Bromide, can reach the nucleus only after the plasma membrane had lost integrity, the color of the nucleus will change from green (when the plasma membranes is still tight, **Supplementary Figure S2E**) over orange to red (**Supplementary Figure S2F**), depending on the degree, to which the plasma membrane is permeabilized. The frequency distributions, constructed over the different concentrations (**Supplementary Figure S2**), show clearly that menthone/isomenthone induced membrane permeability at significantly lower doses and to a significantly higher amplitude (**Supplementary Figure S2A**) as compared to limonene (**Supplementary Figure S2B**), or the essential oil of *A. rugosa* (**Supplementary Figure S2C**). For menthone/isomenthone, from 0.25% (v/v) the Ethidium Bromide label had penetrated into the vast majority of cells within the incubation time of 15 min. The nuclei in those cells displayed a rough and irregular surface and in some cases, there were even indications of blebbing (**Supplementary Figure S2F**, arrow). The fact that compounds that are closely related in terms of structure differ in cytotoxicity, and that the effects of menthone/isomenthone and menthol are enhanced in cells, where microtubules have been mildly stabilised argues for a specific activity of menthone/isomenthone. We, therefore, followed this further focussing on microtubules as potential target.

### Microtubule Disruption in BY2 Cells

**Figure 5:**
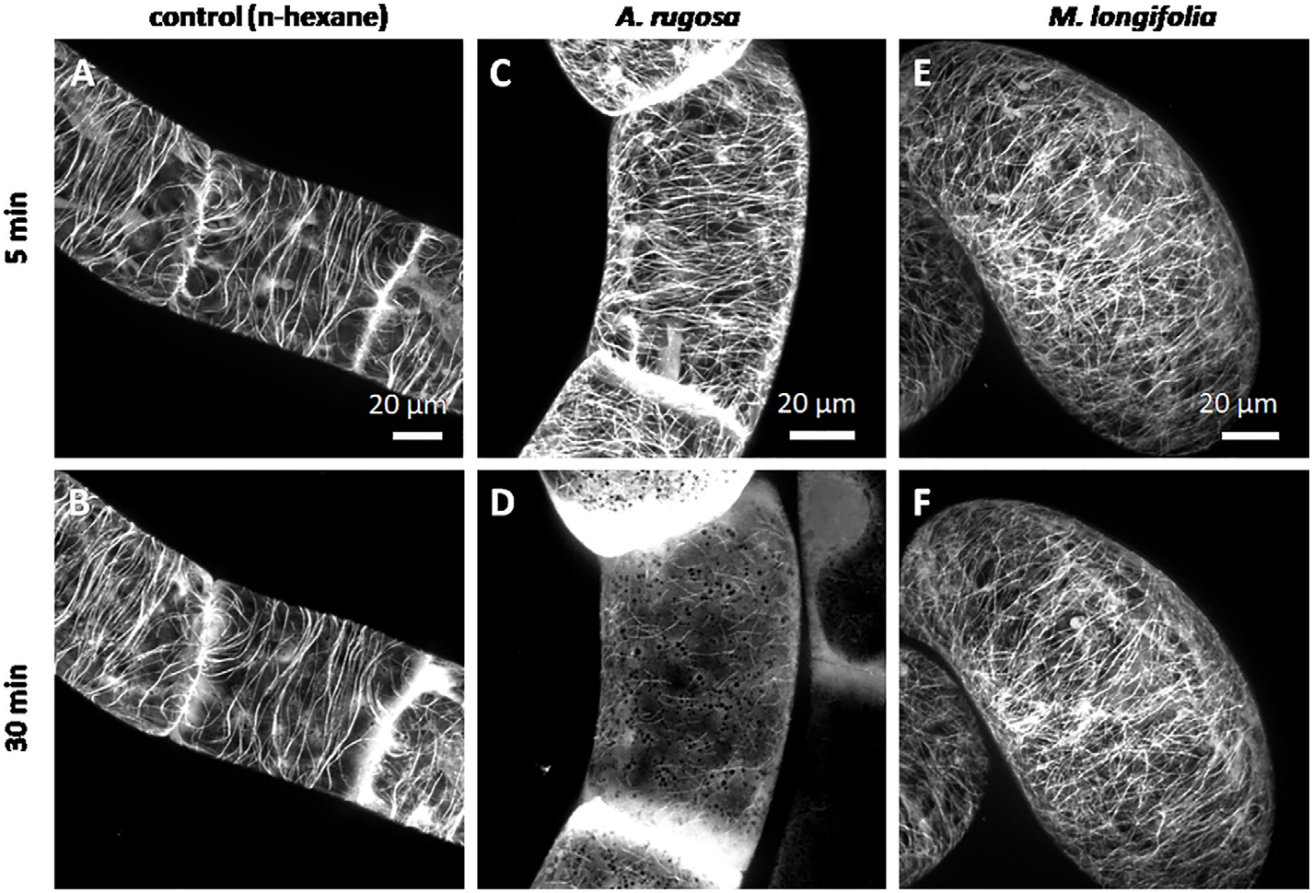
Response of cortical microtubules to essential oils from *A. rugosa* **(C, D)** and *M. longifolia* **(E, F)** in BY-2 cells expressing GFP-TuB6 followed under the spinning disc microscope as compared to the solvent control (n-hexane **A, B**). Cells were treated at day 3 after sub-cultivation.

**Figure 6:**
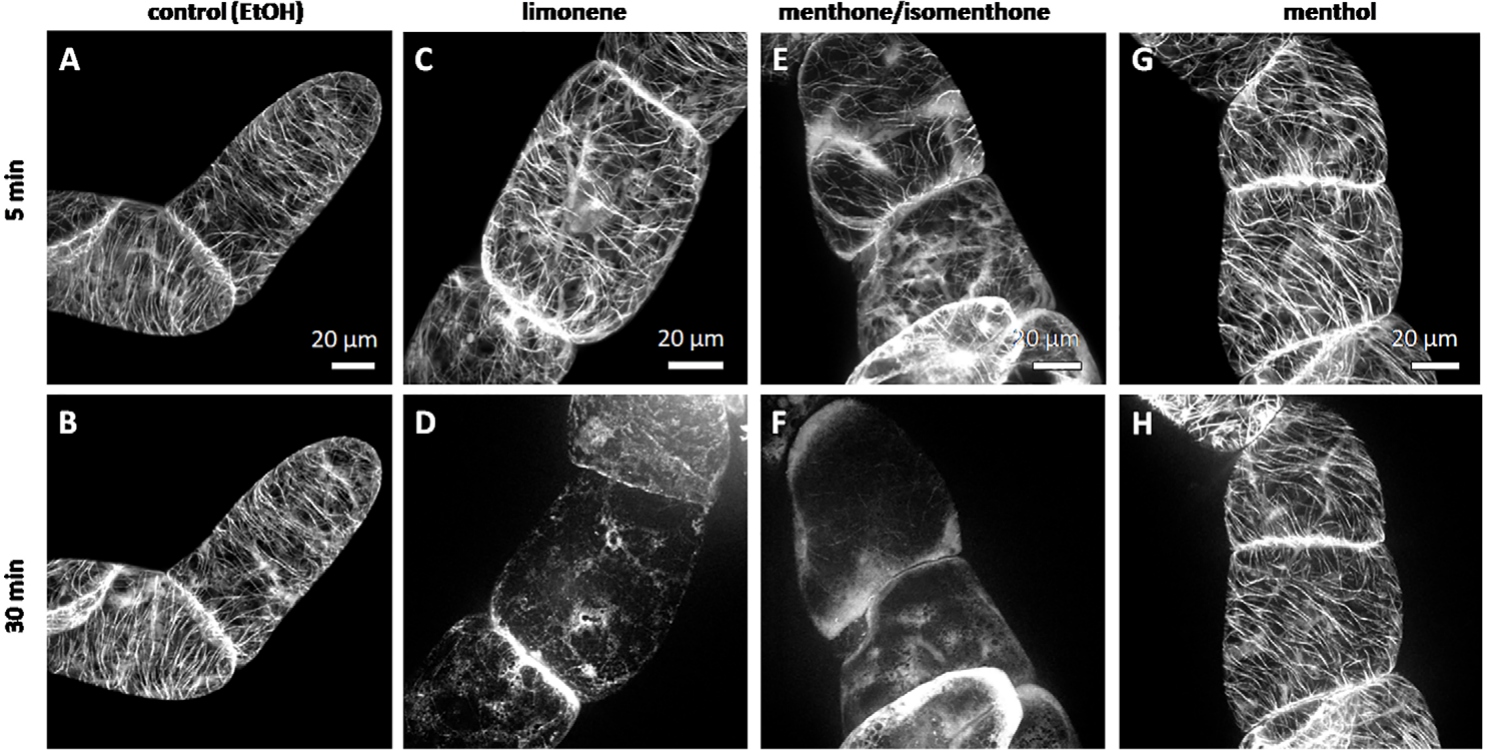
Response of cortical microtubules to limonene **(C, D)**, menthone/isomenthone **(E, F)**, and menthol **(G, H)** in BY-2 cells expressing GFP-TuB6 followed under the spinning disc microscopeas compared to the solvent control ethanol **(A, B)**. Cells were treated at day 3 after subcultivation.

We followed the response of microtubules to the essential oil of *A. rugosa* by spinning-disc confocal microscopy. The tested oils and pure compounds were administered such that they were not in direct contact with the cells, but had to reach their target through the gas phase. Thus, the effective concentrations were much lower than in the mortality assays described before. While the solvent control (n-hexane) did not solicit any microtubule response (**Figures 5A, B**), microtubules were almost completely eliminated after 30 min exposure to the essential oil of *A. rugosa* (**Figures 5C, D**). Only very thin (but still relatively long) remnants of the microtubules were seen, while a diffuse fluorescence was observed in the cytoplasm, and a bright, but diffuse signal accumulated at the cross walls. We also tested the essential oil from *M. longifolia*, which had been found to inhibit germination to a similar extent as the oil from *A. rugosa* (**Figure 2A**). However, here microtubules were not affected (**Figures 5E, F**), but remained intact as in the solvent control. In the next step, we repeated the experiment with menthone/isomenthone, its precursor limonene, and its derivative menthol (**Figure 6**). While microtubules in the solvent control (ethanol) remained intact (**Figures 6A, B**), they were rapidly eliminated by menthone/isomenthone (**Figures 6E, F**). The response was so fast that already at 5 min, the earliest time point that was technically observable, microtubules appeared significantly thinner (**Figure 6E**) as compared to the solvent control (**Figure 6A**). After exposure to menthone/isomenthone for 30 min, only very thin, barely visible, microtubules remained. Similar to the situation for the essential oil from *A. rugosa*, a diffuse background fluorescence was seen in the cytoplasm (**Figure 6F**). Also, the precursor limonene made microtubules disappear (**Figures 6C, D****)**, albeit in a slightly different manner. Here, the signal appeared to coagulate, and there was also no diffuse background fluorescence. In contrast, treatment with menthol did not perturb microtubules (**Figures 6G, H**).

As outgroup for the Lamiaceae, we tested essential oil obtained from *Leptospermum citratum*, which also had been observed to strongly inhibit germination (**Figure 2A**). In fact, this oil also exhibited a strong microtubule-eliminating activity (**Supplementary Figure S1**), and was found to be rich in citral (**Figure 2C**), a compound, for which a strong activity against microtubules had been reported in previous studies (Chaimovitsh et al., 2010; Chaimovitsh et al., 2012). We observed that citral A made microtubules appear thinner as in the control (**Supplementary Figure S1C**), albeit the effect was weaker than that seen for the essential oil from *Leptospermum citratum* (**Supplementary Figure S1B**), and also much milder than the effect evoked by menthone/isomenthone (**Figures 6E, F**).

To test, whether the effect of menthone/isomenthone was specific for microtubules, we also probed the response of actin filaments using tobacco BY-2 cells expressing the actin-binding domain of plant fimbrin. Here, neither for the essential oil of *Agastache rugosa* (**Supplementary Figures S3A, B**), nor for menthone/isomenthone (**Supplementary Figures S3C, D**) any significant elimination of actin filaments was observed, which was in stark contrast to the effect upon microtubules (**Figures 5** and **6**). Actin microfilaments did show a mild response, though, evident as bundling of fine strands into more prominent cables and the emergence of a perinuclear cage of actin after prolonged incubation (**Supplementary Figures S3B, D**).

The results as shown above suggest to the possibility that the differences obtained between the effects caused by different monoterpenes were related to the possible differences in their volatility or solubility.

### Microtubule Disruption in *Arabidopsis thaliana*

**Figure 7:**
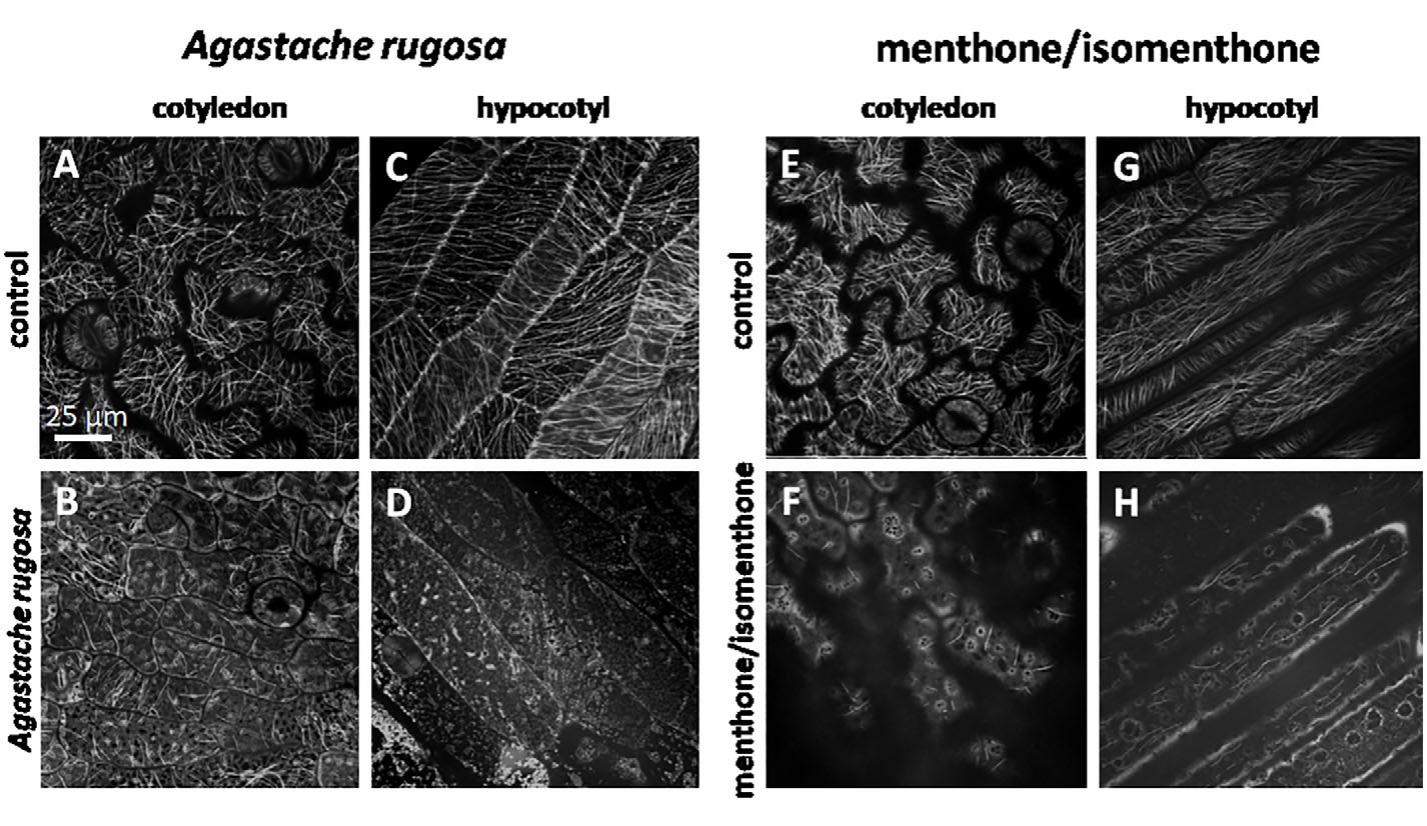
Response of cortical microtubule in etiolated seedlings of *A thaliana* expressing GFP-TuB6 to 50 µl of oil extracted from *A rugosa* **(C, D)**, or menthone/isomenthone **(F, H)** as compared to controls treated with water **(A, C, E, G)** either in epidermal cells of the cotyledon **(A, B, E, F)** or the hypocotyl **(C, D, G, H)**.

To test, whether the effect of *Agastache rugosa* essential oil, or menthone/isomenthone against microtubules is also observed *in planta*, we used transgenic *Arabidopsis thaliana* plants expressing the GFP AtTuB6 marker exposed to either to distilled water (**Figures 7A, B, E, F****)**, the essential oil from *A. rugosa* (**Figures 7C, D**), or to menthone/isomenthone (**Figures 7G, H**), respectively, and observed the response immediately after application by spinning disc confocal microscopy. Then, microtubules were observed in pavement cells of the cotyledon epidermis, and the epidermis of the hypocotyl. Under control conditions, the microtubules in the pavement cells of the cotyledon were clearly visible and organized in parallel bundles that were oriented in different directions, usually more or less transverse to the expansion axis of the respective lobe (**Figures 7A, E**). They were rapidly and thoroughly eliminated by the essential oil from *A. rugosa* (**Figure 7B**), as well as by menthone/isomenthone (**Figure 7F**), whereby menthone/isomenthone was more effective with only very few microtubular remnants persisting the treatment, while with the essential oil, more microtubules remained. Similar to the situation in tobacco BY-2 cells, a soluble, diffuse fluorescence was observed, which accumulated around vesicular structures that partially agglomerated, best visible in the samples treated by menthone/isomenthone (**Figure 7F**). Also in the epidermis of the hypocotyl, microtubules were oriented in parallel arrays that were preferentially transverse in younger, still not fully elongated cells (**Figure 7C**), while in older, fully elongated cells, they were seen more often in longitudinal direction (**Figure 7G**). Again, these microtubules were eliminated almost completely by the essential oil of *A. rugosa* (**Figure 7D**), or menthone/isomenthone (**Figure 7H**). Here, only few microtubules persisted, while, similar to the situation in pavement cells, a diffuse fluorescence surrounding circular or vesicular structures was observed (**Figure 7H**). After treatment with the essential oil from *A. rugosa*, still some microtubules could be observed that displayed a bead-on-a-string pattern indicative of still ongoing disassembly (**Figure 7D**). To test, whether the effect required direct contact with the oil, or the compounds, the experiment was repeated with a set-up, where the oil, or the compounds had to pass through the gas phase (**Supplementary Figure S4B**). Also, in this set-up, the microtubules were disrupted by menthone and the essential oil of *A. rugosa*, while the solvent controls were not effective (**Supplementary Figures S5** and **S6**). To further confirm the effects of the essential oil from *A. rugosa* and menthone/isomenthone, an experiment was setup where seedlings treated with Taxol (providing stability to the microtubules) were treated with the *A. rugosa* essential oil and menthone/isomenthone (**Supplementary Figures S5** and **S6**). Summarizing, the effect of *A. rugosa* essential oil and menthone/isomenthone against microtubules can be clearly confirmed *in planta* as well.

## Discussion

Using a comparative approach, the allelopathic effects of essential oils from different Mints were tested using the standard cress germination assay. Based on the correlation between bioactivity and chemical composition, menthone/isomenthone was identified as a prime candidate for the allelopathic effect and then analyzed further with respect to the mode of action. We can show that menthone/isomenthone causes a rapid elimination of microtubules in both tobacco BY-2 cells and seedlings of *Arabidopsis thaliana*. This effect is specific for microtubules and not seen for actin. It is also specific with respect to structure and function of the compound and much weaker for the structurally closely related menthol. The mortality induced by menthone/isomenthone depends on tubulin abundance since it is accentuated in a BY-2 strain overexpressing a GFP-tagged tubulin as compared to non-transformed cells. The resulting mortality is preceded by permeabilization of the plasma membrane. These key findings lead to several scientific questions, but also bear on future applications. In the following, we will explore the anti-microtubular activity of menthone/isomenthone from different viewpoints: (i) What can we infer on the potential causal relationship with cytotoxicity? (ii) What can we infer on the potential functional context? (iii) What can we infer on the potential molecular mechanism? (iv)What are potentials and challenges of application?

### Microtubules or Membrane—What Is the Primary Target for Menthone/Isomenthone?

The rapid elimination of microtubules (**Figures 6E, F**) in response to menthone/isomenthone is accompanied by a loss of membrane integrity and cell death (**Figure 4**, **Supplementary Figure S2**). This allows two interpretations: the microtubule elimination could act upstream and release a cellular response leading to a unspecific permeabilization of the membrane, or, inversely, menthone/isomenthone could lead to a permeabilization of the membrane that would kill the cells and, secondarily, cause a breakdown of microtubules. There are several arguments against the second scenario: first, the biological effect is specific with respect to structure: the structurally closely related menthol is much less effective as compared to menthone/isomenthone (**Figures 3**, **4**, and **6**), although it is very efficient as membrane permeabilizer (Wang and Meng, 2017). Along the same line, menthone/isomenthone is effective at lower dosage in BY-2 cells overexpressing the TuB6-GFP marker as compared to the non-transformed WT (**Figure 4C**) which speaks against unspecific membrane permeabilization as primary cause for microtubule elimination. Furthermore, under conditions, where microtubules are completely eliminated (**Figures 6E, F**), actin filaments still persist (**Supplementary Figures 3C, D**), which would not be expected, when the cytoskeletal response would be the unspecific consequence of cell death. Furthermore, the response of microtubules seems to occur more swiftly than the permeabilization of the membrane: When the cells were treated with menthone/isomenthone through the gas phase, i.e. at very low effective concentrations, already at the earliest testable time point, 5 min after exposure to menthone/isomenthone, microtubules were significantly affected as compared to the solvent control (**Figure 6**), while with direct contact at much higher concentrations, even after 15 min many cells still had retained a partial membrane integrity (**Supplementary Figure 2A**). Similar to menthol, menthone/isomenthone can permeabilize membranes (Lan et al., 2016), probably interacting with the polar moiety of phospholipids and ceramides, such that a physical contact with microtubules might be conceivable. However, the fact that the microtubule response was observed under conditions, where membrane permeability was retained, speaks rather for a specific binding site at the outer face of the plasma membrane that deploys a cellular response culminating in the disassembly of microtubules. This conclusion is also consistent with results of a study, where a panel of monoterpenes was tested with respect to microtubule degradation (using a transgenic *Arabidopsis *line over-expressing the GFP-tagged alpha tubulin TUA6), and the plasma membrane was assessed using the endocytosis marker FM4-64 (Chaimovitsh et al., 2017). It was found that only Limonene, out of the compounds used in the study, at low concentration was affecting the membrane as shown both by FM4-64 staining and by membrane leakage test.

### Allelopathy Needs Specificity—Some Remarks on Methodology and Functional Context

We used a collection of different Mints as input for this study and found qualitative differences with respect to chemical profile and bioactivity (**Figure 2**), supporting the notion that allelopathic activity acts in a very specific manner. Since Mints belong to the taxonomically more challenging clades of the already challenging family of Lamiaceae, comprising estimated 7,000 species, authentication of the material used for bioactivity screening is mandatory. Moreover, Lindley’s *dictum* that Lamiacean taxonomy with its excessive synonyms and ambiguities became “the disgrace of Botany” (Lindley, 1829), although improved over the last years, still holds valid. We used the genetic barcoding marker *psbA-trnH intergenic spacer*, which for the Lamiaceae genus *Ocimum* was shown to provide better resolution than other markers (Jürges et al., 2018). While it was possible to discern *Mentha* from the other genera, and even several species within the genus, albeit often with low bootstrap support (**Figure 1**), the two species *M. x piperita* and *M. longifolia* were not discriminated. This might be due to hybridization events that would not be detected by a marker located in the plastid genome, such as *psbA-trnH intergenic spacer* (Gobert et al., 2006). This certainly represents a limitation of the otherwise powerful approach to use such organelle markers for genetic barcoding.

Even so, several cases could be detected, where accessions had been incorrectly assigned, which, if it had remained unnoticed, would have the potential to create considerable confusion, since the bioactivity differed considerable between different accessions. For instance, the wrongly assigned *M. suaveolens* produced low allelopathic activity, while the correctly assigned *M. longifolia* was very effective (**Figure 2A**). Unfortunately, the detected cases of wrongly assigned accessions are not a mishap, but a general problem, as demonstrated by a systematic study estimating that around half of tropical species in public collections are mislabelled (Goodwin et al., 2015). If biodiversity is used as input for functional studies such as the current research, this problem has to be addressed. While genetic barcoding has acquired attention as powerful strategy to authenticate (for a recent review see Thakur et al., 2019), any barcode is only as good as the reference plant is authentic. Unfortunately, this aspect is often neglected, when sequences collected from commercial samples are deposited in public databases without any further authentication of the material. Again, a recent systematic study has found that considerable proportions (ranging from around 25% in Asia up to 80% in Australia) of commercial herbal products were adulterated (Ichim, 2019). This will create considerable spill-over of wrongly assigned sequence information in public databases. We have, therefore, for good reasons, used only curated sequence information, where the database entry gave some information about species identification and voucher material, and we strongly recommend that this should become common practice in any work involving genetic barcoding. Thus, the quality of genetic barcoding depends on the care, by which the respective specimen has been taxonomically identified. There is clearly a bottleneck, as accentuated in the conclusion by Goodwin et al. (2015): *“Rapidly increasing numbers of specimens in an increasing numbers of herbaria are not being revised because there are too few taxonomists.”*. Molecular methods, as powerful as they are, do not rescue us from this bottleneck.

Putting aside these methodological considerations, the pronounced species dependency of the allelopathic effect leads to some interesting biological questions: When allelopathy is used as tool to outcompete other species (possibly even from the same genus—some of the investigated Mints do occur sympatrically), there must be a mechanism that prevents self-inhibition of the donor. In case of a non-specific cellular activity, this would only be achieved by controlling the spread of the compound in space. While this seems to be the case for the production, contained to the excretion cavity of glandular hairs and scales, this is certainly not true once the essential oil is released. If, however, the effect of the compounds is linked to the presence of specific binding sites at the membrane of the receiver cell, the donor could protect itself by mutating or protecting this binding site and thus reducing it sensitivity. Thus, identification and functional analysis for the menthone/isomenthone binding site represents a crucial issue to get deeper insight into the functional context of this allelopathic interaction.

### Ideas on the Microtubular Basis of Cytotoxicity

Even among the chemically diverse Lamiaceae, the Mints excel by their rich chemical diversity, where even accessions from the same species differ with respect to the composition of their essential oils (Kokkini, 1991). Using this diversity as platform for an activity-guided fractionation approach, we identified menthone/isomenthone as compound with a strong inhibitory effect on seed germination, possibly responsible for the strong effect of *A. rugosa* essential oil. This finding is compatible with previous work, where menthone has been identified as major component in *A. rugosa* (Zielińska et al., 2017), and was found to exert a moderate inhibition of germination in a comparative study of different monoterpenes (Martino et al., 2010), albeit at very high concentrations and only in radish, while not effective in cress. The inhibitory effect of *A. rugosa* essential oil and menthone/isomenthone correlated well with an elimination of microtubules, seen both, in tobacco BY-2 cells as well as in different tissues of *Arabidopsis* seedlings. This effect was specific for microtubules and not seen for actin filaments, which is reminiscent for the effect of the monoterpene citral (Chaimovitsh et al., 2010; Chaimovitsh et al., 2012). We have, therefore, included two species of Lemon Myrtle (*Backhousia citriodora, Leptospermum citratum*) as positive controls into the study, because the essential oils from these species are rich in citral (**Figure 2C**), and we were able to see a strong inhibition of germination (**Figure 2A**), and a strong elimination of microtubules (**Supplementary Figure S1B**), as to be inferred from the Chaimovitsh et al. (2010). The elimination of microtubules is accompanied by the increase of diffuse background signal in the cytoplasm, presumably representing non-polymerised tubulin heterodimers. This was observed both, for the essential oil of *A. rugosa* (**Figure 5D**), as for menthone/isomentone (**Figure 6F**), and indicates that microtubules are not disrupted, but that the assembly or elongation of microtubules is impaired such that microtubules are eliminated due to their innate turnover. Again, this is reminiscent to the mode of action of citral: here, the association of γ-tubulin, an essential component of microtubule nucleation was disrupted (Chaimovitsh et al., 2012), as well as the assembly of microtubules *in vitro* (Chaimovitsh et al., 2010). As discussed above, the elimination of microtubules seems to be responsible for the subsequent cell death and loss of membrane integrity. However, the mechanism linking microtubule elimination to cell death is still unknown, but the elevated sensitivity of the BY-2 strain overexpressing TuB6-GFP, as compared to the non-transformed wild type, indicates that it is the excess of non-polymerised tubulin heterodimers that triggers cell death. This would be in line with a phenomenon observed all over the eukaryotes, where overexpression of tubulins causes lethality (yeast: Katz et al., 1990; maize: Anthony and Hussey, 1998). In fact, the steady-state levels of tubulin heterodimers are regulated very tightly by both, post-translational, and transcriptional control circuits (for review see Breviario and Nick, 2000). We are currently pursuing the hypothesis, whether metacaspases, components of the machinery executing programmed cell death (Gong et al., 2019) might be activated depending on their association with microtubules.

### Outlook: Potentials and Challenges of New Bioherbicides

Weeds are one major reason for yield losses—for instance, just for India alone the damage caused by weeds is estimated to range at around 4.4 billion US$ per year (Gharde et al., 2018). While herbicides had been a central asset for the success of the so called Green Revolution, there has been growing concern about the hazardous effects of synthetic herbicides, and the problem of herbicide resistance (for a classical review see Pimentel, 1996). As a result, the spectrum of lead structures for plant-protection control has been trimmed down, such that new mode of actions, and the use of compounds of natural origin have shifted into attention (Dayan et al., 2012). Alellochemicals with their pronounced specificity and their innate function to suppress competitors represent promising candidates for the search of bioherbicides (for comprehensive reviews see Singh et al., 2003; Macias et al., 2007; Soltys et al., 2013). We have used the pronounced allelopathy of *Mentha* to identify menthone/isomenthone in a bio-activity-guided strategy.

Microtubules provide a promising target for several groups of herbicide including the phenyl carbamates, or the dinitroanilines, such as trifluralin or oryzalin (reviewed in Vaughn, 2000). This mode of action has certain drawbacks, though: tubulin is a fairly conservative molecule, and a compound interfering with plant microtubules is likely to interfere with animal microtubules as well, such that these compounds would be potentially cancerogenic or mutagenic. However, these herbicides acting against microtubules target to molecular moieties, where plant and animal tubulins differ, such as the C-terminus of α-tubulin targeted by phenylcarbamates (Wiesler et al., 2002), or an amino-acid substitution in the N-terminal region of α-tubulin that allows the binding of dinitroanilines in plants, but not in animals (Anthony et al., 1998). A second strategy to obtain plant-specific microtubule elimination would be not to target tubulin directly, but to target the signalling that culminates in microtubule elimination. Signalling in plant cells is fundamentally different, and there is plenty of room to find specific targets that do not occur in animal cells. This is the strategy, which we propose in the current work. There are still several tasks to be solved to render menthone/isomenthone or the essential oil of *A. rugosa* into a tool applicable as bioherbicide. One problem is the volatility—on the one hand, this property allows for efficient spread, on the other hand, it will cause rapid decay of the pool that has been administered to the field. Here, we are currently testing different slow-release carriers under conditions that are close to agricultural application.

## Data Availability Statement

The datasets presented in this study can be found in online repositories. The names of the repository/repositories and accession number(s) can be found below:

https://www.ncbi.nlm.nih.gov/, MH753578.1

https://www.ncbi.nlm.nih.gov/, MH753577.1

https://www.ncbi.nlm.nih.gov/, MH753576.1

https://www.ncbi.nlm.nih.gov/, MH753575.1

https://www.ncbi.nlm.nih.gov/, MH753574.1

https://www.ncbi.nlm.nih.gov/, MH753573.1

https://www.ncbi.nlm.nih.gov/, MH753572.1

https://www.ncbi.nlm.nih.gov/, MH753571.1

https://www.ncbi.nlm.nih.gov/, MH753570.1.

## Author Contributions

MS: performed experiments, interpretation of data, writing. FR: performed experiments, interpretation of data. MK: performed experiments, interpretation of data. WB: performed experiments, interpretation of data. SW: interpretation of data, writing. KM: performed experiments. AK: performed experiments, interpretation of data, writing. VS: designing of experiment, interpretation of data, writing. PN: design of experiment, interpretation of data, writing, supervision of project.

## Conflict of Interest

The authors declare that the research was conducted in the absence of any commercial or financial relationships that could be construed as a potential conflict of interest.

## Supplementary Material

Supplementary Figure 1 Response of cortical microtubules to essential oils from *L citratum* **(B)** and citral A **(C)** in BY-2 cells expressing GFP-TuB6 followed under the spinning disc microscope as compared to the solvent control (n-hexane, A). Cells were treated at day 3 after subcultivation.

Supplementary Figure 2 Mortality of BY2 cells after treatment with menthone/isomenthone, limonene and oil extracted from *A rugosa*, characterized by differential cellular staining. Frequency distribution of stages of cell death after treatment with menthone/isomenthone **(A)**, limonene **(B)**, and essential oil from *A. rugosa* **(C)**. The color code shows different stages of cell death ranging from living cells to dead cells. **(D)** Control cells stained with a combination of Acridine Orange and Ethidium Bromide shows intact plasma membrane. **(E)** Disintegration of plasma membrane after treatment with 0.25% v/v menthone/isomenthone. **(F)** Nuclei with perturbed shape after treatment with menthone/isomenthone, after double staining with Acridine Orange and Ethidium Bromide.

Supplementary Figure 3 Response of actin filaments to essential oil from *A. rugosa* **(A, B)** or menthone/isomenthone **(C, D)** for either 5 min **(A, C)**, or 30 min **(B, D)**, respectively. Inaction was visualized by the actin-binding domain of plant fimbrin and followed under the spinning disc microscope. Cells were treated at day 3 after sub-cultivation.

Supplementary Figure 4 Diagrammatic representation of the treatment of **(A)** BY2 cells, and **(B)** *Arabidopsis thaliana* seedlings. The white circles represent the droplets of essential oils or compounds, respectively. The arrows indicate the diffusion across the gas phase, when cover slip is placed on the samples

Supplementary Figure 5 Response of cortical microtubules in etiolated seedlings of *A thaliana* expressing GFP TuB6 to compounds administered through the gas phase. Plants were grown on ½ MS agar plates supplemented with 1% w/v sucrose for 5 d in the the dark at 25°C. The seedlings exposed to either a gas phase of 50 µl n-hexane as a solvent control **(B)**, menthone/isomenthone **(C)** or oil extracted from *A rugosa* **(D)** were compared to control **(A)**. Plants were treated in 10 µM Taxol (Paclitaxel) for 1 h (E, I, K) before exposure to either the gas phase from 50 µl of *n*-hexane **(F)**, menthone/isomenthone **(G, J)** or oil extracted from *A. rugosa* **(H, L)** either in epidermal pavement cells of the cotyledon **(A–H)** or the shoot apical meristem **(I–L)**. The time interval between mounting and observation was 5 min. Scale Bar is of 25 µm.

Supplementary Figure 6 The seedlings exposed to either a gas phase of 50 µl n-hexane as a solvent control **(B)**, menthone/isomenthone **(C)**, or oil extracted from *A rugosa* **(D)** were compared to control **(A)**. Plants were treated in 10 µM Taxol (Paclitaxel) for 1 h **(E)** before exposure to either the gas phase from 50 µl of *n*-hexane **(F)**, menthone/isomenthone **(G)** or oil extracted from *A. rugosa* **(H)** in epidermal cells of the hypocotyl. The time interval between mounting and observation was 5 min. Scale Bar is of 25 µm.

Supplementary Data S1 Heat map for bioactivity and abundance of compounds in the oils extracted from the aromatic plants used in this study. The bioactivity and the abundance of compounds as shown in the heat map shows the high bioactivity of menthone/isomenthone and also their high presence in *A. rugosa* and *M. longifolia*.
