# Supplementary figures and images for: Cellular Base of Mint Allelopathy: Menthone Affects Plant Microtubules
Source: Front Plant Sci. 2020 Sep 16;11:546345. doi: 10.3389/fpls.2020.546345 (PMC7524878; doi:10.3389/fpls.2020.546345)

## Slide 1
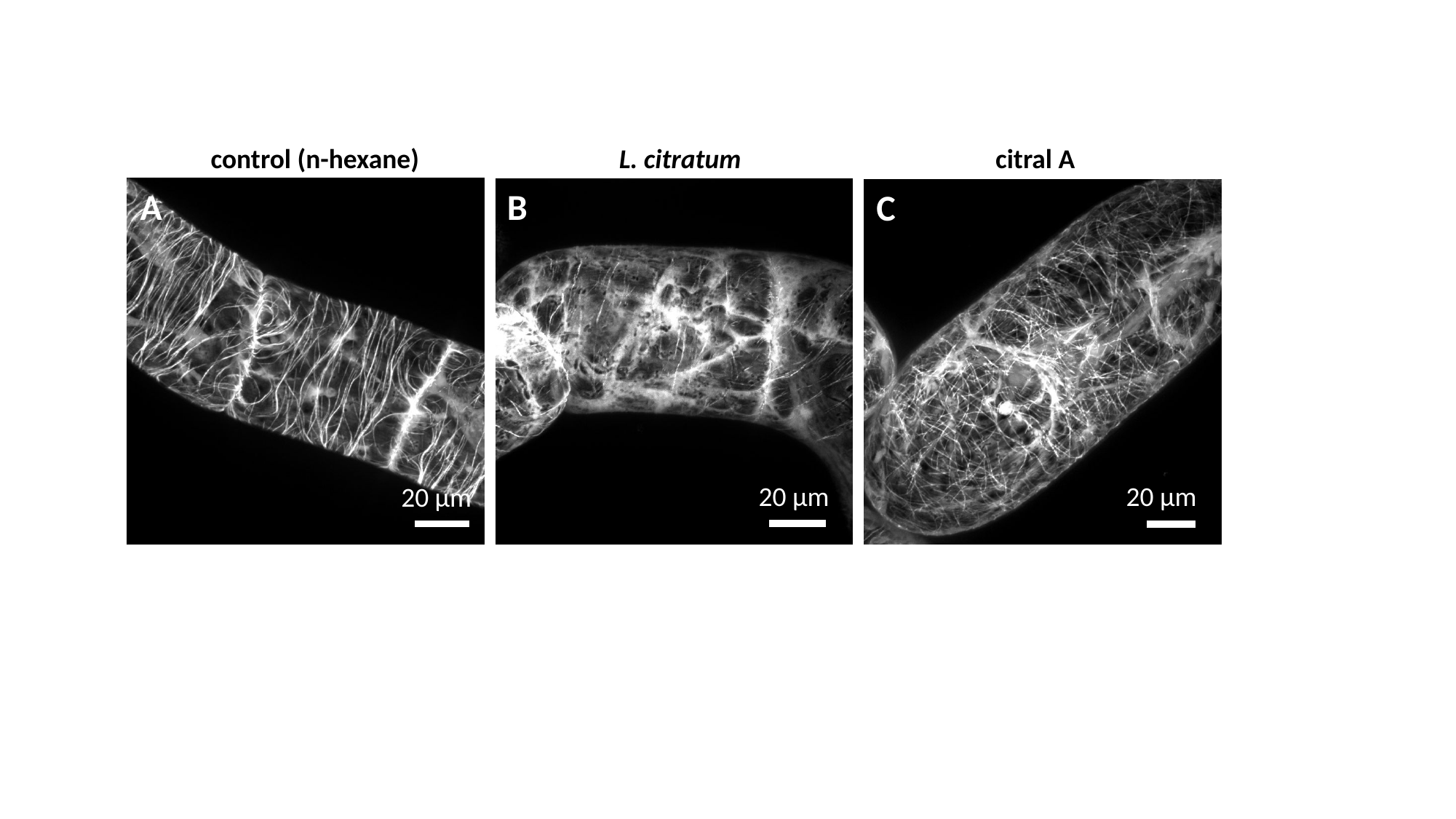

citral A
L. citratum
control (n-hexane)
B
A
C
20 µm
20 µm
20 µm

Supplement: Supplementary Figure 1 — Response of cortical microtubules to essential oils from L citratum (B) and citral A (C) in BY-2 cells expressing GFP-TuB6 followed under the spinning disc microscope as compared to the solvent control (n-hexane, A). Cells were treated at day 3 after subcultivation. [file Presentation_1.pptx]

## Slide 1
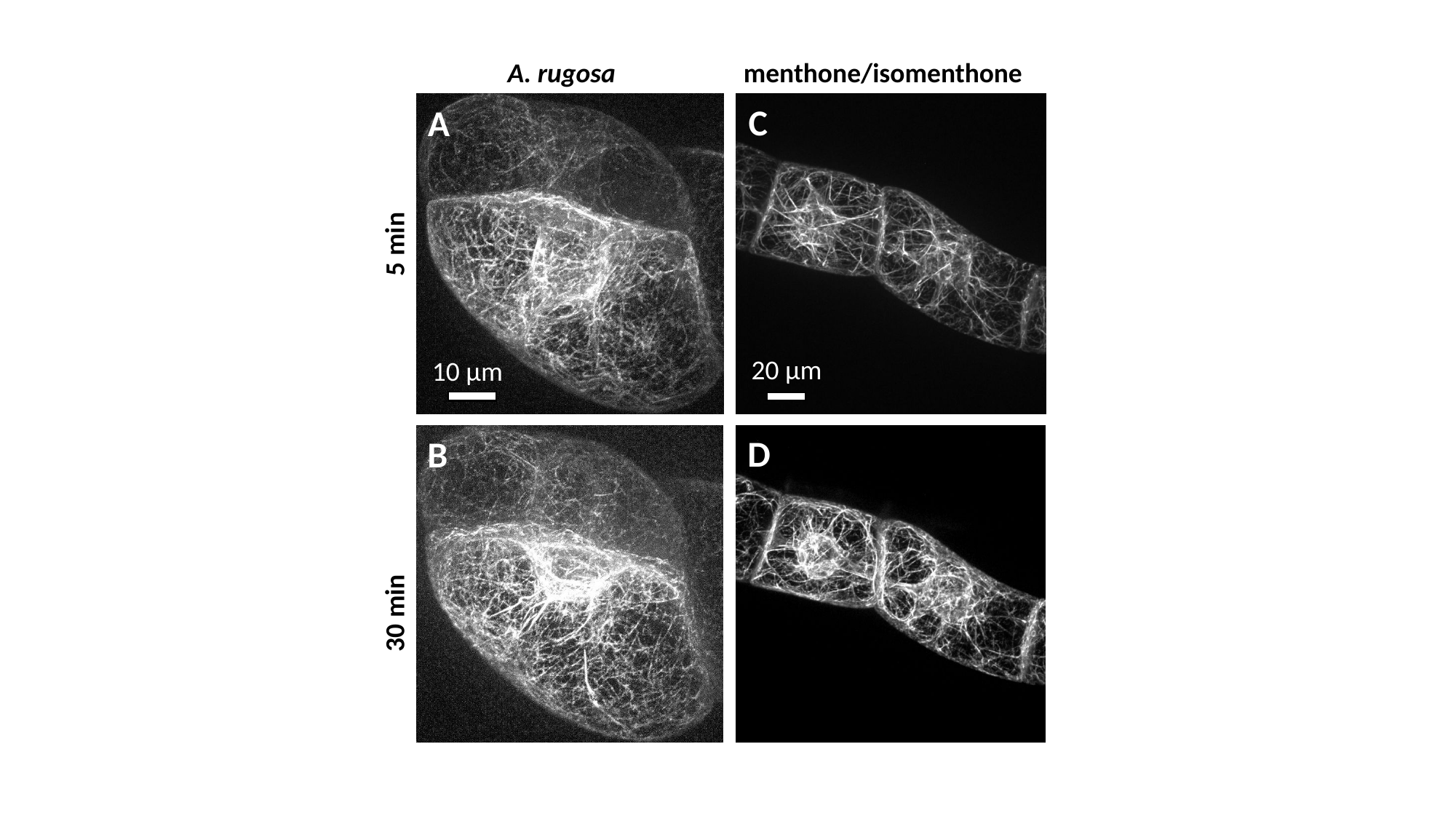

A. rugosa
menthone/isomenthone
C
A
5 min
20 µm
10 µm
D
B
30 min

Supplement: Supplementary Figure 3 — Response of actin filaments to essential oil from A. rugosa (A, B) or menthone/isomenthone (C, D) for either 5 min (A, C), or 30 min (B, D), respectively. Inaction was visualized by the actin-binding domain of plant fimbrin and followed under the spinning disc microscope. Cells were treated at day 3 after sub-cultivation. [file Presentation_3.pptx]
