# Supplementary material for: Cellular Base of Mint Allelopathy: Menthone Affects Plant Microtubules
Source: Front Plant Sci. 2020 Sep 16;11:546345. doi: 10.3389/fpls.2020.546345 (PMC7524878; doi:10.3389/fpls.2020.546345)
Supplement: Supplementary Figure 2 — Mortality of BY2 cells after treatment with menthone/isomenthone, limonene and oil extracted from A rugosa, characterized by differential cellular staining. Frequency distribution of stages of cell death after treatment with menthone/isomenthone (A), limonene (B), and essential oil from A. rugosa (C). The color code shows different stages of cell death ranging from living cells to dead cells. (D) Control cells stained with a combination of Acridine Orange and Ethidium Bromide shows intact plasma membrane. (E) Disintegration of plasma membrane after treatment with 0.25% v/v menthone/isomenthone. (F) Nuclei with perturbed shape after treatment with menthone/isomenthone, after double staining with Acridine Orange and Ethidium Bromide. [file Presentation_2.pptx]

## Slide 1
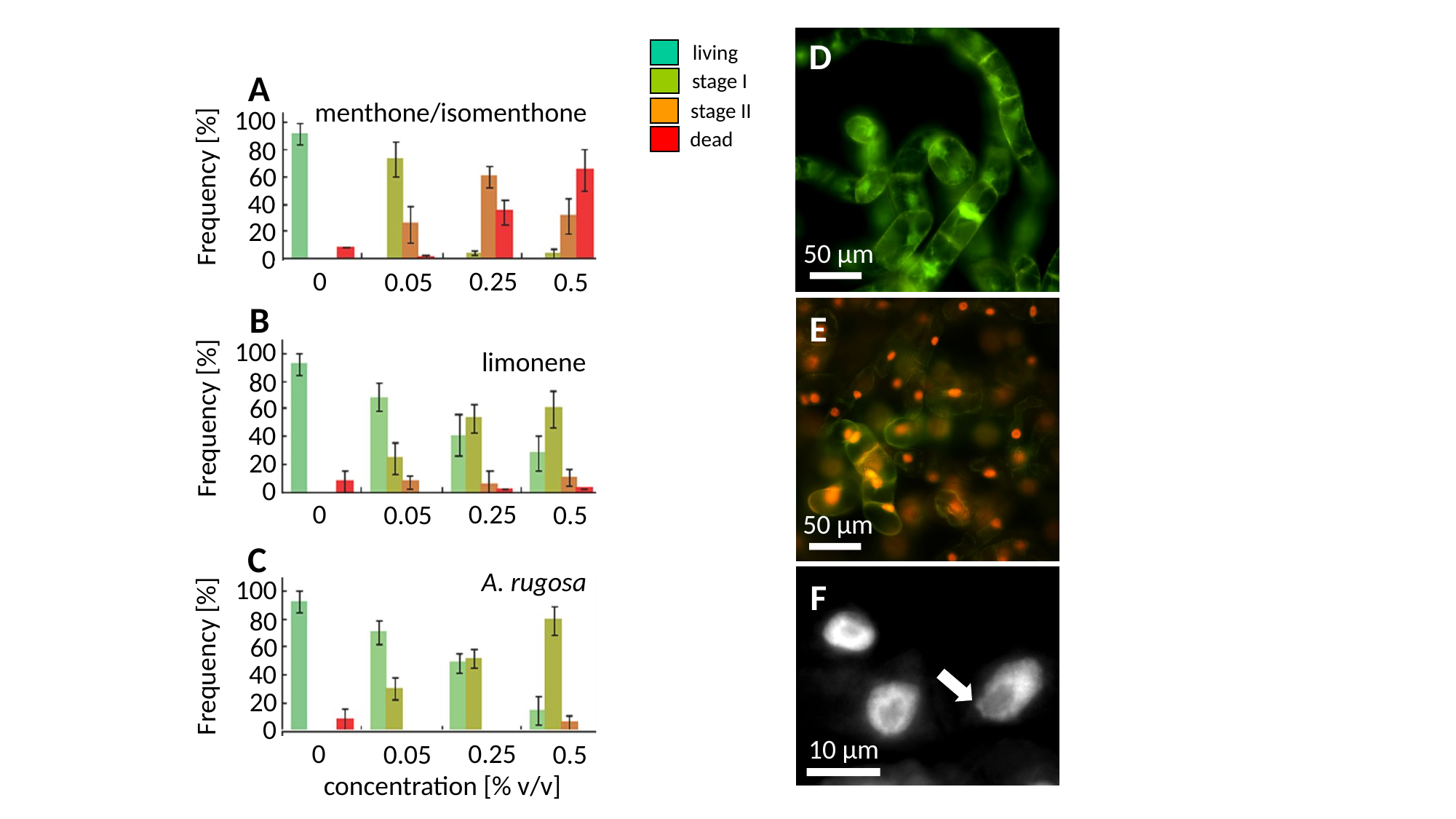

D
living
A
stage I
menthone/isomenthone
stage II
100
dead
80
60
Frequency [%]
40
20
50 µm
0
0
0.25
0.5
0.05
B
E
100
limonene
80
60
Frequency [%]
40
20
0
0
0.25
0.5
0.05
50 µm
C
A. rugosa
100
F
80
60
Frequency [%]
40
20
0
10 µm
0
0.25
0.5
0.05
concentration [% v/v]
