# Supplementary material for: Cellular Base of Mint Allelopathy: Menthone Affects Plant Microtubules
Source: Front Plant Sci. 2020 Sep 16;11:546345. doi: 10.3389/fpls.2020.546345 (PMC7524878; doi:10.3389/fpls.2020.546345)
Supplement: Supplementary Figure 4 — Diagrammatic representation of the treatment of (A) BY2 cells, and (B) Arabidopsis thaliana seedlings. The white circles represent the droplets of essential oils or compounds, respectively. The arrows indicate the diffusion across the gas phase, when cover slip is placed on the samples [file Presentation_4.pptx]

## Slide 1
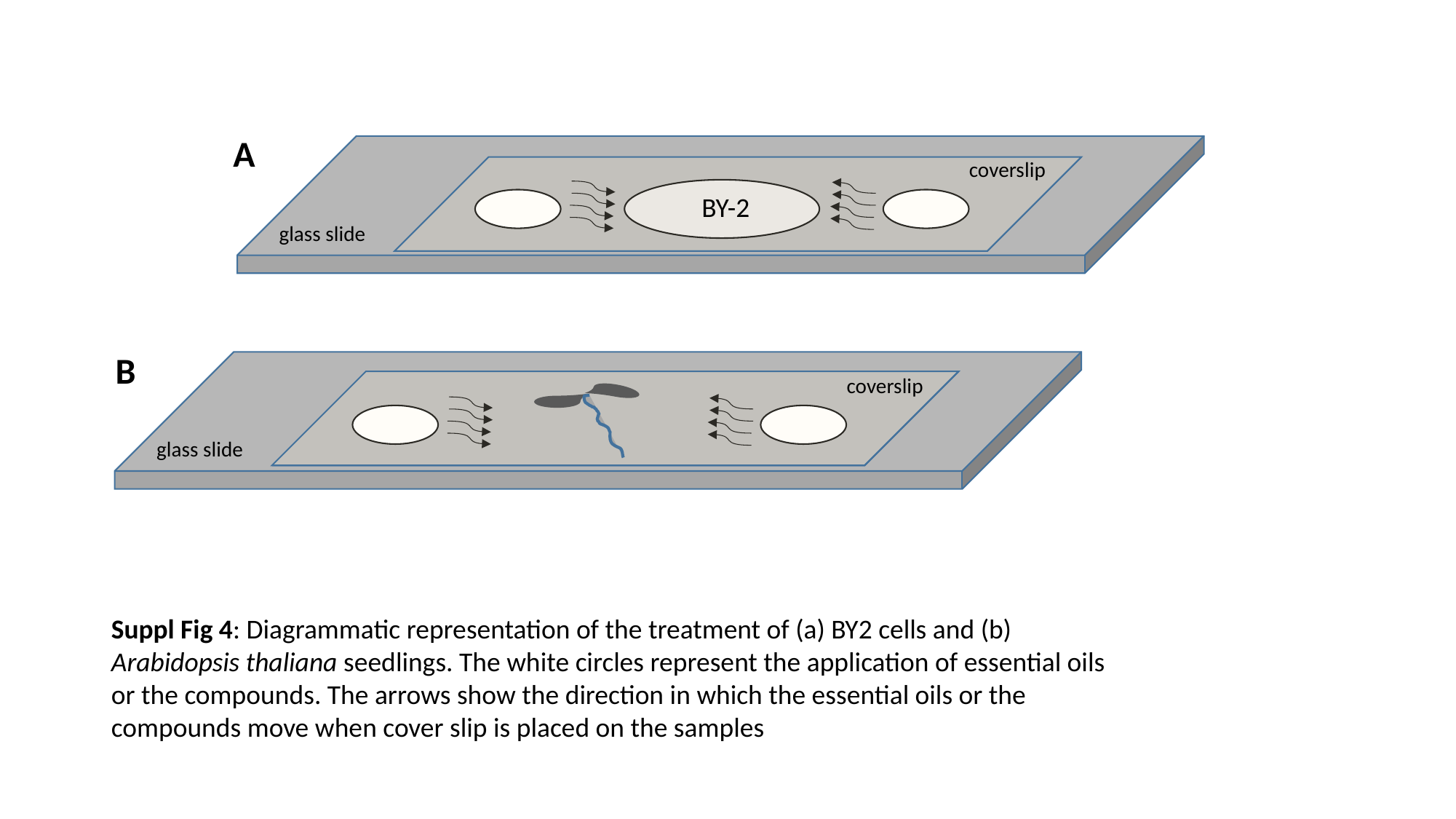

A
coverslip
BY-2
glass slide
B
coverslip
glass slide
Suppl Fig 4: Diagrammatic representation of the treatment of (a) BY2 cells and (b) Arabidopsis thaliana seedlings. The white circles represent the application of essential oils or the compounds. The arrows show the direction in which the essential oils or the compounds move when cover slip is placed on the samples
