# Supplementary material for: Cellular Base of Mint Allelopathy: Menthone Affects Plant Microtubules
Source: Front Plant Sci. 2020 Sep 16;11:546345. doi: 10.3389/fpls.2020.546345 (PMC7524878; doi:10.3389/fpls.2020.546345)
Supplement: Supplementary Figure 5 — Response of cortical microtubules in etiolated seedlings of A thaliana expressing GFP TuB6 to compounds administered through the gas phase. Plants were grown on ½ MS agar plates supplemented with 1% w/v sucrose for 5 d in the the dark at 25°C. The seedlings exposed to either a gas phase of 50 µl n-hexane as a solvent control (B), menthone/isomenthone (C) or oil extracted from A rugosa (D) were compared to control (A). Plants were treated in 10 µM Taxol (Paclitaxel) for 1 h (E, I, K) before exposure to either the gas phase from 50 µl of n-hexane (F), menthone/isomenthone (G, J) or oil extracted from A. rugosa (H, L) either in epidermal pavement cells of the cotyledon (A–H) or the shoot apical meristem (I–L). The time interval between mounting and observation was 5 min. Scale Bar is of 25 µm. [file Presentation_5.pptx]

## Slide 1
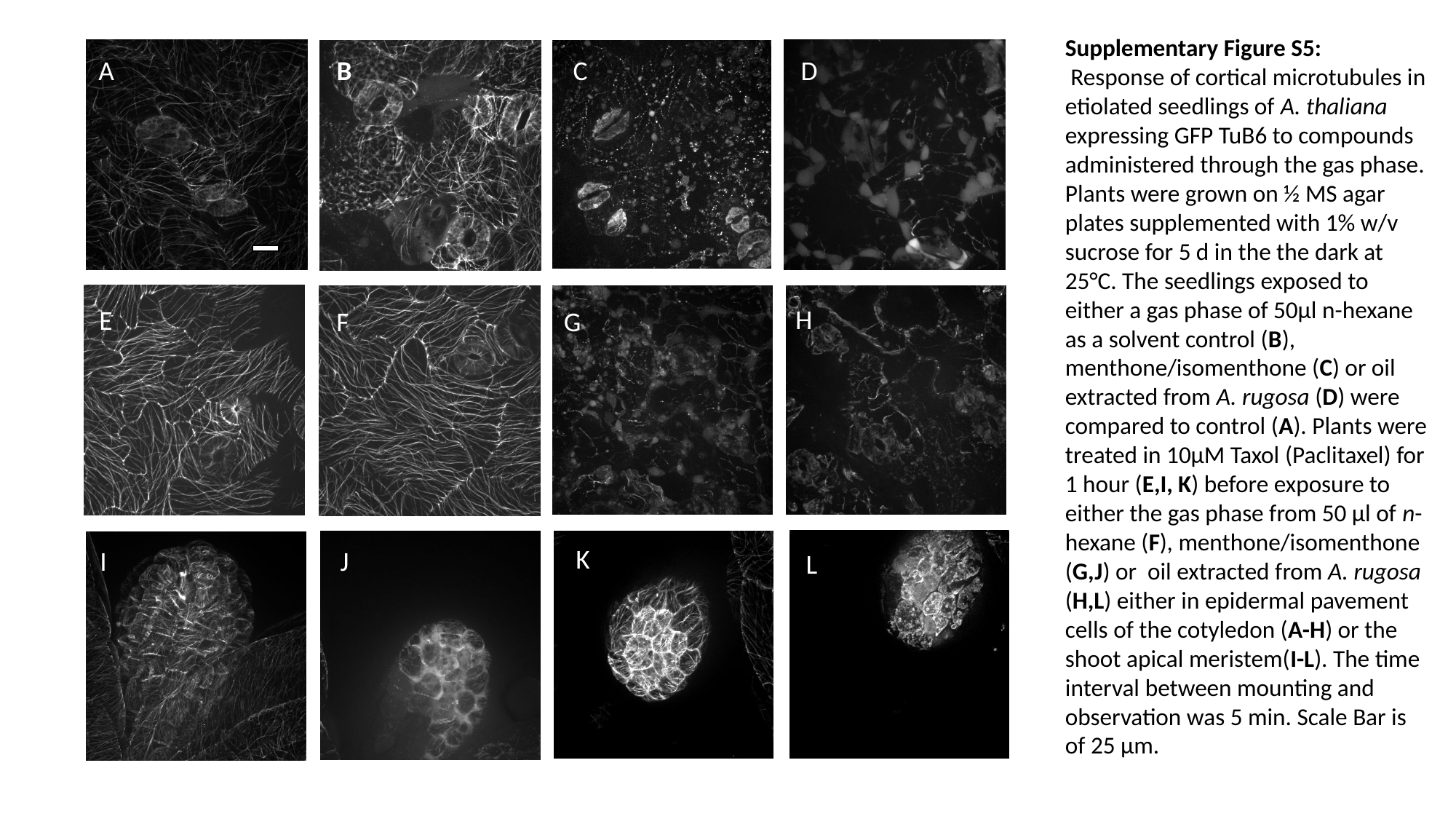

Supplementary Figure S5:
 Response of cortical microtubules in etiolated seedlings of A. thaliana expressing GFP TuB6 to compounds administered through the gas phase. Plants were grown on ½ MS agar plates supplemented with 1% w/v sucrose for 5 d in the the dark at 25°C. The seedlings exposed to either a gas phase of 50µl n-hexane as a solvent control (B), menthone/isomenthone (C) or oil extracted from A. rugosa (D) were compared to control (A). Plants were treated in 10µM Taxol (Paclitaxel) for 1 hour (E,I, K) before exposure to either the gas phase from 50 µl of n-hexane (F), menthone/isomenthone (G,J) or oil extracted from A. rugosa (H,L) either in epidermal pavement cells of the cotyledon (A-H) or the shoot apical meristem(I-L). The time interval between mounting and observation was 5 min. Scale Bar is of 25 µm.
A
B
C
D
H
E
G
F
K
I
J
L
