# Supplementary material for: Cellular Base of Mint Allelopathy: Menthone Affects Plant Microtubules
Source: Front Plant Sci. 2020 Sep 16;11:546345. doi: 10.3389/fpls.2020.546345 (PMC7524878; doi:10.3389/fpls.2020.546345)
Supplement: Supplementary Figure 6 — The seedlings exposed to either a gas phase of 50 µl n-hexane as a solvent control (B), menthone/isomenthone (C), or oil extracted from A rugosa (D) were compared to control (A). Plants were treated in 10 µM Taxol (Paclitaxel) for 1 h (E) before exposure to either the gas phase from 50 µl of n-hexane (F), menthone/isomenthone (G) or oil extracted from A. rugosa (H) in epidermal cells of the hypocotyl. The time interval between mounting and observation was 5 min. Scale Bar is of 25 µm. [file Presentation_6.pptx]

## Slide 1
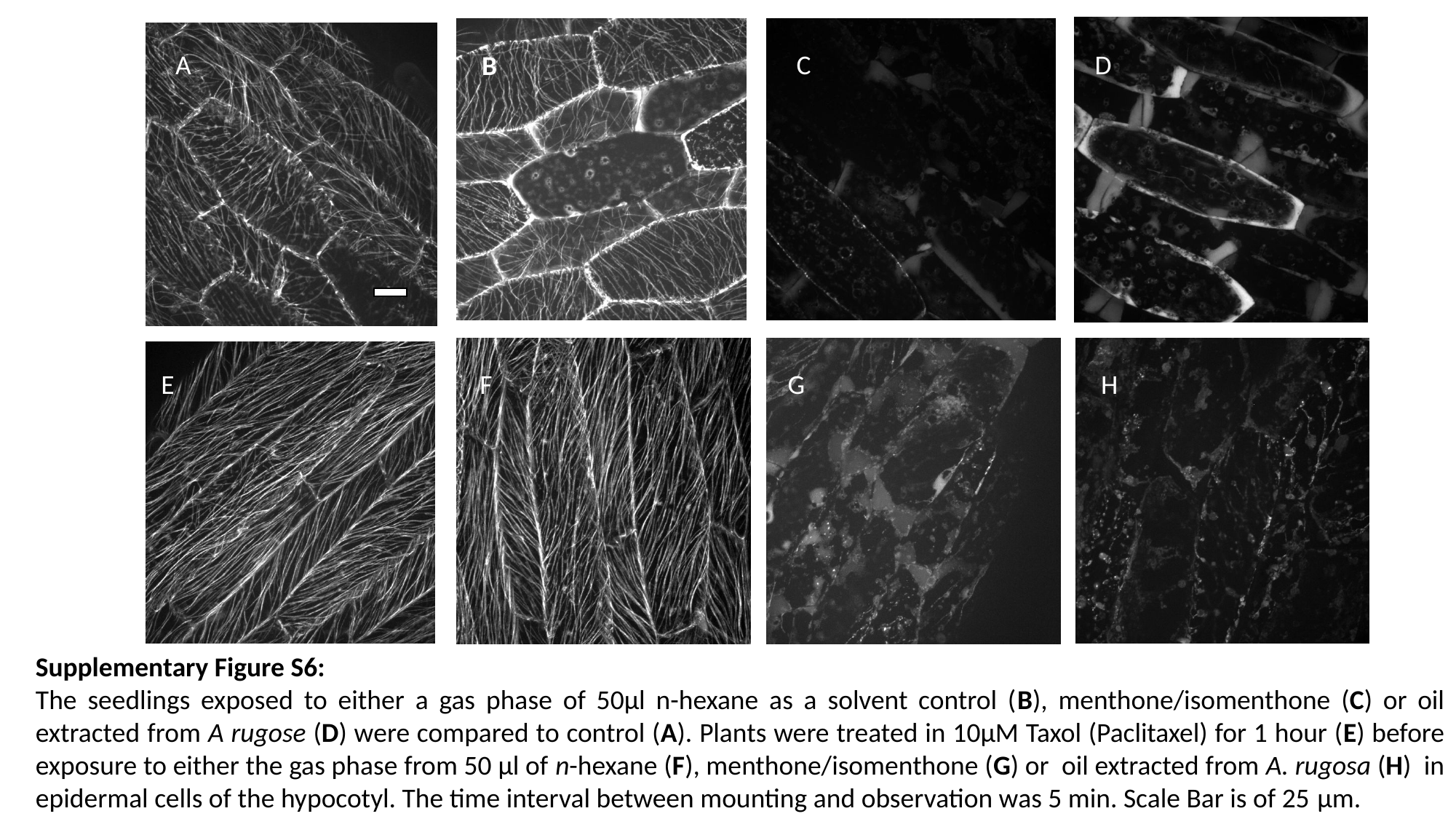

A
C
D
B
E
F
G
H
Supplementary Figure S6:
The seedlings exposed to either a gas phase of 50µl n-hexane as a solvent control (B), menthone/isomenthone (C) or oil extracted from A rugose (D) were compared to control (A). Plants were treated in 10µM Taxol (Paclitaxel) for 1 hour (E) before exposure to either the gas phase from 50 µl of n-hexane (F), menthone/isomenthone (G) or oil extracted from A. rugosa (H) in epidermal cells of the hypocotyl. The time interval between mounting and observation was 5 min. Scale Bar is of 25 µm.
