# Supplementary material for: Cellular Base of Mint Allelopathy: Menthone Affects Plant Microtubules
Source: Front Plant Sci. 2020 Sep 16;11:546345. doi: 10.3389/fpls.2020.546345 (PMC7524878; doi:10.3389/fpls.2020.546345)
Supplement: Supplementary Data S1 — Heat map for bioactivity and abundance of compounds in the oils extracted from the aromatic plants used in this study. The bioactivity and the abundance of compounds as shown in the heat map shows the high bioactivity of menthone/isomenthone and also their high presence in A. rugosa and M. longifolia. [file DataSheet_1.pdf]

colour code % abundance

| Colour | % abundance |
|---|---|
| light blue | <1% |
| light yellow | 1-5% |
| light orange | 5-10% |
| orange | 10-25% |
| pink | 25-50% |
| red | >50% |

colour code bioactivity

| Colour | bioactivity |
|---|---|
| blue | <50 |
| green | 50-100 |
| yellow | 100-200 |
| orange | 200-500 |
| red | >500 |

bioactivity using 0.1 ppm of essential oil

| | M_spicata | M_spicata_cri | M_suaveolen | M_longifolia | M_piperita | A_rugosa | N_cataria | M_officinalis | L_citratum | B_citriodora | Bioactivity score | |
|---|---|---|---|---|---|---|---|---|---|---|---|---|
| inhibition [%] | 84.3 | 84.7 | 23.5 | 81.3 | 25.4 | 79.6 | 72.6 | 0.42 | 93.4 | 97.3 | | |
| se | 4.5 | 3.7 | 2.8 | 2.1 | 6.2 | 5.9 | 4.8 | 0.2 | 0.7 | 0.7 | | |
| | M_spicata | M_spicata_cri | M_suaveolen | M_longifolia | M_piperita | A_rugosa | N_cataria | M_officinalis | L_citratum | B_citriodora | | |
| alpha pinene | 0.15 | 0.23 | 0.24 | 0.21 | 0.26 | 0 | 0 | 0 | 0 | 0 | 6.14 | alpha pinene |
| beta pinene | 0.5 | 0.86 | 0.49 | 0.69 | 0.72 | 0.07 | 0 | 0 | 0 | | 20.65 | beta pinene |
| 1-octene-3-ol | 0 | 0 | 0.66 | 0 | 0 | 0.97 | 0 | 0.1 | 0 | | 9.28 | 1-octene-3-ol |
| beta myrcene | 1.23 | 1.57 | 0.32 | 0.06 | 0.33 | 0.56 | 0 | 0.26 | 1.41 | 0.22 | 45.52 | beta myrcene |
| limonene | 5.46 | 12.99 | 0.64 | 0 | 0 | 9.58 | 0 | 0 | 0 | 0 | 233.81 | limonene |
| beta-ocimene | 0 | 0.66 | 0.4 | 0.12 | 0.85 | 0 | 0 | 0 | 0.04 | 0 | 10.04 | beta-ocimene |
| eucalyptol | 0.24 | 0 | 0 | 0 | 0 | 0 | 0 | 0 | 0.06 | | 2.58 | eucalyptol |
| sabinene | 0 | 0 | 0 | 1.72 | 0 | 0 | 0 | 0 | 0 | 0 | 13.98 | sabinene |
| linalool | 0 | 0 | 0 | 0.07 | 0 | 0 | 0 | 0.08 | 1.92 | 0.27 | 21.13 | linalool |
| iso-pulegone | 0 | 0 | 0 | 0.05 | 0 | 0 | 0 | 0.14 | 4.52 | 0.19 | 44.48 | iso-pulegone |
| menthone | 0.27 | 0 | 0 | 19.37 | 0 | 4.58 | 0 | 0 | 0 | 0 | 196.21 | menthone |
| citronellal | 0 | 0 | 0 | 0 | 0 | 0 | 0 | 0.65 | 36.22 | 0.09 | 339.20 | citronellal |
| menthone+isomenthone | 0.27 | 0.07 | 0 | 28.19 | 0 | 36.47 | 0 | 0.07 | 0 | 0 | 522.36 | menthone+iso-menthone |
| iso-menthone | 0 | 0.07 | 0 | 8.82 | 0 | 31.89 | 0 | 0.07 | 0 | 0 | 326.15 | iso-menthone |
| iso-neral | 0 | 0 | 0 | 0 | 0 | 0 | 0 | 0 | 0.71 | 1.04 | 16.75 | iso-neral |
| menthol | 0 | 0 | 0.3 | 53.54 | 0 | 0.57 | 0 | 0 | 0.01 | 0 | 440.62 | menthol |
| isogeranial | 0 | 0 | 0 | 0.9 | 0 | 0 | 0 | 0.58 | 0.74 | 1.58 | 29.63 | isogeranial |
| alpha-terpineol | | 0.05 | 0 | 0.3 | 0.38 | 0.02 | 0 | 0 | 0 | 0 | 3.99 | alpha-terpineol |
| dihydro-carvone | 0.43 | 0.44 | 0 | 0 | 0 | 0 | 0 | 0 | 0 | 0 | 7.35 | dihydro-carvone |
| trans-carveol | 0 | 0.29 | 0 | 0 | 0 | 0.03 | 0 | 0.02 | 0 | 0 | 2.70 | trans-carveol |
| beta-citronellol | 0 | 0 | 0.05 | 0 | 0 | 0.02 | 0 | 0.03 | 1.83 | 0.04 | 17.76 | beta-citronellol |
| cis-hexenyl-isovalerate | 0 | 0 | 0 | 0 | 0.16 | 0 | 0 | 0 | 0 | 0 | 0.41 | cis-hexenyl-isovalerate |
| pulegone | 0 | 0 | 0 | 0 | 0 | 47.23 | 0 | 0 | 0 | 0 | 375.95 | pulegone |
| carvone | 78.91 | 70.45 | 0.09 | 0 | 0 | 0 | 0 | 0 | 0 | 0 | 1262.13 | carvone |
| beta-citral | 0 | 0 | 0 | 0 | 0 | 0 | 0 | 30 | 20.36 | 42.42 | 604.17 | beta-citral |
| piperitone-epoxid | 0 | 0 | 55.51 | 0.32 | 77.65 | 0.03 | 0 | 0 | 0 | 0 | 330.52 | piperitone-epoxid |
| geraniol | 0 | 0 | 0 | 0 | 0 | 0 | 0 | 0 | 1.82 | 0.13 | 18.26 | geraniol |
| alpha-citral | 0 | 0 | 0.08 | 0 | 0 | 0 | 0 | 42.97 | 26.28 | 0 | 247.45 | alpha-citral |
| epoxy-linalooloxid | 0 | 0 | 0 | 0 | 0.24 | 0 | 0 | 7.49 | 0 | 0 | 0.92 | epoxy-linalooloxid |
| piperitone oxid | 0 | 0 | 23.62 | 0 | 5.88 | 0 | 0 | 0 | 0 | 0 | 70.44 | piperitone oxid |
| geranic acid | 0 | 0 | 0 | 0 | 0 | 0 | 0 | 5.59 | 0 | 0 | 0.23 | geranic acid |
| beta-bourbonene | 2.59 | 1.07 | 0.17 | 0.22 | 0 | 0.04 | 0 | 0.55 | 0.11 | 0 | 34.45 | beta-bourbonene |
| beta-elemene | 0.69 | 0 | 0.06 | 0 | 0 | 0 | 0.77 | 0 | 0.21 | 0 | 13.51 | beta-elemene |
| beta-caryophyllene | 1.72 | 1.07 | 0 | 1.18 | 3.39 | 0.7 | 1.07 | 0 | 0.14 | 0 | 56.41 | beta-caryophyllene |
| beta-copaene | 0.16 | 0 | 0 | 0 | 0 | 0 | 0 | 0.07 | 0 | 0 | 1.35 | beta-copaene |
| cadina-3,5-diene | 0.14 | 0 | 1.19 | 0 | 0 | 0 | 0 | 0 | 0 | 0 | 3.98 | cadina-3,5-diene |
| alpha-humulene | 0.03 | 0 | 0.81 | 0 | 0 | 0 | 0 | 0 | 0.01 | 0 | 2.25 | alpha-humulene |
| cis-muurola-4(15),5-diene | 0 | 0.3 | 2.44 | 0.16 | 0.78 | 0 | 0.16 | 0.04 | 0.02 | 0 | 12.91 | cis-muurola-4(15),5-diene |
| germacrene D | 1.77 | 2.73 | 7.47 | 2.69 | 4.32 | 0.42 | 0 | 0 | 0.02 | 0 | 91.97 | germacrene D |
| bicyclo-germacrene | 0.04 | 0.33 | 0.08 | 0.4 | 0.19 | 0.28 | 1.52 | 0 | 0.6 | 0 | 25.92 | bicyclo-germacrene |
| germacrene A | 1.32 | 0 | 0.16 | 0.03 | 0.03 | 0 | 0 | 0.04 | 0.26 | 0 | 14.25 | germacrene A |
| trans-calamenene | 0.02 | 0.09 | 0.62 | | 0.06 | 0 | 0 | 0 | 0 | 0 | 2.54 | trans-calamenene |
| delta-cadinene | 0 | 0 | 0 | | 0 | 0.09 | 0 | 0 | 0 | 0 | 0.72 | delta-cadinene |
| caryophyllene-oxid | 0 | 0 | 0 | 0 | 0 | 0 | 0.43 | 4.79 | 0 | 0 | 3.32 | caryophyllene-oxid |
| 1-epi-cubenol | 0 | 0.12 | 0.86 | 0 | 0 | 0 | 0 | 0 | 0 | 0 | 3.04 | 1-epi-cubenol |
| alpha-cadenol | 0 | 0 | 0.54 | 0 | 0.05 | 0.25 | 0 | 0 | 0.23 | 0 | 5.53 | alpha-cadenol |
| 6a-Hydroxygermacra-1(10 | 0.02 | 0.07 | 0.02 | 0 | 0 | 0 | 0 | 0 | 0 | 0 | 0.81 | 6a-Hydroxygermacra-1(10),4-diene |
