# Supplementary material for: Cellular Base of Mint Allelopathy: Menthone Affects Plant Microtubules
Source: Front Plant Sci. 2020 Sep 16;11:546345. doi: 10.3389/fpls.2020.546345 (PMC7524878; doi:10.3389/fpls.2020.546345)
Supplement: Supplementary file 8 [file Table_1.docx]

**Table 1:** Accessions used in the current study maintained as living specimens in the Botanical Garden of the Karlsruhe Institute of Technology under the indicated voucher ID. Scientific taxonomy follows the The Plant List ([www.theplantlist.org](http://www.theplantlist.org)). All *psbA-trnH* *igs* sequences from these accessions were deposited in GenBank under the given ID and identity verified by comparing these accessions with curated sequences from GenBank (see **Fig. 1**).

| KIT ID | Declared taxonomy | Vernacular names | Source | GenBank ID for *psbA-trnH* *igs* | Identity |
| --- | --- | --- | --- | --- | --- |
| 7579 | *Mentha spicata* L. | Spear Mint | BG KIT | MH753576 | *M. spec.* |
| 5391 | *Mentha spicata* L. *var. crispa* | Curly Mint | BG KIT | MH753570 | *M. spicata* L. |
| 8680 | *Mentha aquatica* L. | Water Mint | WEL 3/451 | MH753578 | *M. aquatica* L. |
| 8681 | *Mentha arvensis* L. | Corn Mint | WEL 3/437 | MH753577 | *M. spec.* |
| 8682 | *Mentha longifolia* (L.) L. | Horse Mint | WEL 3/72 | MH753572 | *M longifolia* (L.) L. |
| 5393 | *Mentha x piperita* L. | Pepper Mint | BG KIT | MH753571 | *M. x piperita* (L.) |
| 3638 | *Mentha suaveolens* Ehrh. | Apple Mint | BG Vakratot | MH753574 | *M. spec.* |
| 4643 | *Nepeta cataria* L. | Catnip | BG KIT | MH753573 | *N. cataria* L. |
| 4639 | *Melissa officinalis* L: | Common Balm | BG KIT | MH781964 | *M. officinalis* L: |
| 7576 | *Agastache rugosa* (Fisch. & C.A.Mey.) Kuntze | Korean Mint | BG KIT | MH753575 | *A. rugosa* (Fisch. & C.A.Mey.) Kuntze |
