# Supplementary material for: Cellular Base of Mint Allelopathy: Menthone Affects Plant Microtubules
Source: Front Plant Sci. 2020 Sep 16;11:546345. doi: 10.3389/fpls.2020.546345 (PMC7524878; doi:10.3389/fpls.2020.546345)
Supplement: Supplementary file 9 [file Table_2.docx]

**Table 2:** oligonucleotide primers used for genetic identification of the tested accessions.

| Name | 5' → 3' sequence | Target | Reference |
| --- | --- | --- | --- |
| *psbA^u^* | GTTATGCATGAACGTAATGCTC | psbA-trnH intergenic spacer | Sang *et al*. (1997) |
| *trnH^u^* | CGCGCATGGTGGATTCACAATCC |  | Tate and Simpson (2003) |
