# Supplementary material for: Cellular Base of Mint Allelopathy: Menthone Affects Plant Microtubules
Source: Front Plant Sci. 2020 Sep 16;11:546345. doi: 10.3389/fpls.2020.546345 (PMC7524878; doi:10.3389/fpls.2020.546345)
Supplement: Supplementary file 10 [file Table_3.docx]

**Table 3:** purity and source information for the monoterpene compounds used in the current study.

| **compound** | **purity (%)** | **source** | **catalogue number** |
| --- | --- | --- | --- |
| Geraniol | analytical standard | Sigma-Aldrich | 163333 |
| β-Citronellol | 95 | Sigma-Aldrich | C83201 |
| α-Pinene | 98 | Sigma-Aldrich | 147524 |
| β-Pinene | pure | Roth | 7106.1 |
| Linalool | 97 | Sigma-Aldrich | L2602 |
| Menthone | analytical standard | Merk | [95401](https://www.sigmaaldrich.com/catalog/product/sial/95401?lang=de&region=DE) |
| DL-Menthol | analytical standard | Sigma-Aldrich | 05174 |
| R-(+)-Limonene | analytical standard | Sigma-Aldrich | 62118 |
